# A fast algorithm to factorize high-dimensional tensor product matrices used in genetic models

**DOI:** 10.1093/g3journal/jkae001

**Published:** 2024-01-05

**Authors:** Marco Lopez-Cruz, Paulino Pérez-Rodríguez, Gustavo de los Campos

**Affiliations:** Department of Epidemiology and Biostatistics, Michigan State University, East Lansing, MI 48824, USA; Socioeconomía, Estadística e Informática, Colegio de Postgraduados, Montecillos, Edo. de México 56230, Mexico; Department of Epidemiology and Biostatistics, Michigan State University, East Lansing, MI 48824, USA; Department of Statistics and Probability, Michigan State University, East Lansing, MI 48824, USA; Institute for Quantitative Health Science and Engineering, Michigan State University, East Lansing, MI 48824, USA

**Keywords:** R package, genetic model, covariance matrix, eigenvalue decomposition

## Abstract

Many genetic models (including models for epistatic effects as well as genetic-by-environment) involve covariance structures that are Hadamard products of lower rank matrices. Implementing these models requires factorizing large Hadamard product matrices. The available algorithms for factorization do not scale well for big data, making the use of some of these models not feasible with large sample sizes. Here, based on properties of Hadamard products and (related) Kronecker products, we propose an algorithm that produces an approximate decomposition that is orders of magnitude faster than the standard eigenvalue decomposition. In this article, we describe the algorithm, show how it can be used to factorize large Hadamard product matrices, present benchmarks, and illustrate the use of the method by presenting an analysis of data from the northern testing locations of the G × E project from the Genomes to Fields Initiative (*n* ∼ 60,000). We implemented the proposed algorithm in the open-source “tensorEVD” R package.

## Introduction

Hadamard products of positive definite matrices appear in many genetic models including gene-by-gene (e.g. additive-by-additive or additive-by-dominance, Henderson 1985) and gene-by-environment interactions (Crossa *et al.* 2006) as well as in hybrid prediction models (Bernardo 1998). In this article, we focus on high-dimensional Hadamard products derived from 2 positive semidefinite matrices, each with a dimension considerably smaller than the resulting Hadamard product.

To motivate this problem, consider a reaction norm infinitesimal model (Falconer and Mackay 1996) for $n_{G}$ genotypes tested over $n_{E}$ locations (environments). Following Jarquín *et al.* (2014), interactions between genetic and environmental factors can be modeled using a Gaussian random effect with a covariance matrix K that is the product of a genetic ($K_{G}$, derived from DNA or pedigree data) and an environmental ($K_{E}$, typically derived from environmental covariates) relationship matrix. If all genotypes are tested in all environments, K is a Kronecker product $K=K_{G}⊗K_{E}$ of dimension $n=n_{G}\times n_{E}$. However, usually, not all genotypes are tested in all environments and genotypes may be replicated. In these cases, the K matrix takes the form $K=(Z_{1}K_{G}Z_{1}^{\prime })\circ (Z_{2}K_{E}Z_{2}^{\prime })$ where $Z_{1}$ and $Z_{2}$ are incidence matrices connecting phenotypes with the rows (and columns) of $K_{G}$ and $K_{E}$, respectively, and “∘” denotes the Hadamard product. A very similar problem arises when modeling hybrids’ effects where $K_{G}$ and $K_{E}$ are replaced by additive relationship matrices between the female and male parental lines (Bernardo 1998).

Fitting Gaussian models with dense covariance structures such as the one presented above requires factorizing K using, for example, the eigenvalue decomposition (EVD) of **K**. The EVD has an $O(n^{3})$ computational complexity; therefore, a standard decomposition of **K** does not scale well to large sample sizes. To tackle this problem, we use results about the EVD of Kronecker products, and the fact that Hadamard products are submatrices of Kronecker products, to propose an algorithm that derives a basis for K that only requires factorizing $K_{G}$ and $K_{E}$ matrices that usually are much smaller than K. We show that the proposed approach provides a very good approximation to the target matrix (K) and that, in large *n* problems, the proposed approach can be orders of magnitude faster than performing EVD on K directly. Finally, we provide real data analyses showing that the proposed approach yields very close variance component estimates and almost an identical prediction accuracy in cross-validation to an exact EVD. The methods described in this article are implemented in the open-source “tensorEVD” R package, which is available through CRAN and the GitHub repository.

## Methods

Recall the EVD of an N×N positive semidefinite matrix K that has the form


$$K\;=VDV^{\prime },$$

where $V=[v_{1},\ldots ,v_{N}]$ is an orthonormal matrix (i.e. V′V=I) whose columns $v_{k}$ (k=1,…,N) are the eigenvectors and $D=\mathrm{diag}(d_{1},\ldots ,d_{N})$ is a diagonal matrix with the eigenvalues $d_{1}\geq \ldots \geq d_{N}\geq 0$.

Consider the Kronecker product (“⊗”) of 2 symmetric positive semidefinite matrices, $K_{1}$ and $K_{2}$,


(1) $$K=K_{1}⊗K_{2}.$$

Let the EVD of the 2 matrices in the right-hand side be $K_{1}=V_{1}D_{1}V_{1}^{\prime }$ and $K_{2}=V_{2}D_{2}V_{2}^{\prime }$, respectively. Replacing these matrices with their EVD we get:


$$K=(V_{1}D_{1}V_{1}^{\prime })⊗(V_{2}D_{2}V_{2}^{\prime }).$$

Using properties of Kronecker products (e.g. Searle 1982, p. 265), it can be shown that the eigenvectors of K are Kronecker products of the eigenvectors of $K_{1}$ and $K_{2}$. Likewise, the eigenvalues of K are Kronecker products of the eigenvalues of $K_{1}$ and $K_{2}$ (see Supplementary Note 1 for a proof). Specifically, we have that (a numerical example of the above results is presented in Supplementary Note 2):


$$K=VDV^{\prime }=(V_{1}⊗V_{2})(D_{1}⊗D_{2}){\left(V_{1}⊗V_{2}\right)}^{\prime }.$$

A Hadamard product (“∘”) of 2 matrices is a submatrix of the corresponding Kronecker product. For example, an n×n matrix:


(2) $$K_{0}=(Z_{1}K_{1}Z_{1}^{\prime })\circ (Z_{2}K_{2}Z_{2}^{\prime }),$$

is a submatrix of $K_{1}⊗K_{2}$ in Equation (1). Therefore, the linear space spanned by $\left(Z_{1}K_{1}Z_{1}^{\prime })\circ (Z_{2}K_{2}Z_{2}^{\prime }\right)$ in Equation (2) is a subspace of the linear space spanned by $K_{1}⊗K_{2}$. This suggests that we can find a basis for a Hadamard product from the EVD of the corresponding Kronecker product. The tensor EVD algorithm is inspired by this idea.

### Tensor EVD algorithm

We assume that the input data consist of the following:

- *Covariance structures*: $K_{1}$ and $K_{2}$ of dimensions $n_{1}\times n_{1}$ and $n_{2}\times n_{2}$, respectively. For example, $K_{1}$ may be a genomic relationship matrix and $K_{2}$ may be an environmental relationship matrix describing environmental similarity between testing environments.
- *IDs*: $ID_{1}$ and $ID_{2}$ are *n* vectors (*n* here is the sample size) mapping from observations to the rows and columns of $K_{1}$ and $K_{2}$, respectively. (The row and column names of $K_{1}$ and $K_{2}$ are the unique entries of ${ID}_{1}$ and ${ID}_{2}$, respectively.) These IDs are used to form the incidence matrices $Z_{1}$ and $Z_{2}$ in Equation (2). For instance, the matrix $Z_{1}K_{1}Z_{1}^{\prime }$ can be obtained by indexing rows and columns of $K_{1}$ by ${ID}_{1}$; in R's (R Core Team 2021) notation, this is $K_{1}[{ID}_{1},{ID}_{1}]$.

Using the above-described inputs, our algorithm (which we named *tensorEVD*) proceeds as follows:

1. Perform the EVD of $K_{1}=V_{1}D_{1}V_{1}^{\prime }$ and $K_{2}=V_{2}D_{2}V_{2}^{\prime }$.
2. Derive the $N=n_{1}\times n_{2}$ eigenvalues of the Kronecker product as $\tilde{D}=\mathrm{diag}({\tilde{d}}_{1},\ldots ,{\tilde{d}}_{N})=D_{1}⊗D_{2}$.
3. Derive the *N* eigenvectors $\tilde{V}=[{\tilde{v}}_{1},\ldots ,{\tilde{v}}_{N}]$ of the Kronecker product. Each column ${\tilde{v}}_{k}$ (k=1,…,N) is the Hadamard product of the $i_{k}^{\mathrm{th}}$ and $j_{k}^{\mathrm{th}}$ eigenvectors of $V_{1}$ and $V_{2}$, respectively, that is ${\tilde{v}}_{k}=(Z_{1}v_{1i_{k}})\circ (Z_{2}v_{2j_{k}})$. As before, the terms $Z_{1}v_{1i_{k}}$ and $Z_{2}v_{2i_{k}}$ are obtained using indexing, i.e. $v_{1i_{k}}[ID_{1}]$ and $v_{2j_{k}}[ID_{2}]$.
4. For unbalanced or replicated data, the eigenvectors in $\tilde{V}$ may not have a norm equal to 1; thus, the sum of the eigenvalues ${\tilde{d}}_{k}$ will no longer be equal to trace(K). Therefore, we normalize each eigenvector ${\tilde{v}}_{k}$ to have unit norm.
5. Order the eigenvalues ${\tilde{d}}_{k}$ and eigenvectors ${\tilde{v}}_{k}$ according to ${\tilde{d}}_{k}$.

The *tensorEVD* algorithm described above renders orthonormal vectors only for the balanced case (i.e. for the Kronecker product of $K_{1}$ and $K_{2}$). For unbalanced cases, the eigenvectors are not guaranteed to be mutually orthogonal; however, they provide a basis for the Kronecker product. Therefore, the eigenvectors are also a basis for Hadamard products that span a subspace of the corresponding Kronecker product.

Note that the *tensorEVD* algorithm produces the complete basis containing $N=n_{1}\times n_{2}\;$ eigenvectors for the Kronecker matrix product $K_{1}⊗K_{2}$. As consequence, this basis can include more vectors than the ones needed to span $\left(Z_{1}K_{1}Z_{1}^{\prime })\circ (Z_{2}K_{2}Z_{2}^{\prime }\right)$. This can be particularly relevant if the size of the Hadamard product is considerably smaller than the corresponding Kronecker product. Furthermore, most of those vectors will have a very small eigenvalue (resulting from the product of a small eigenvalue of $K_{1}$ and a small eigenvalue from $K_{2}$). Therefore, instead of forming all possible eigenvectors, we allow for the user to specify a proportion of variance explained (0<α≤1, e.g. α=0.95) and build only the eigenvectors needed to achieve such proportion of variance.

The “tensorEVD” R package can be installed from CRAN using the following instruction:

**Table:** 

install.packages(‘tensorEVD’)

Alternatively, it can be installed from the GitHub platform via, for instance, the “remotes” R package (Csárdi *et al*. 2023) using the instructions below:

**Table:** 

install.packages(‘remotes’) library(remotes) install_github(‘MarcooLopez/tensorEVD’)

The following script shows how to perform EVD using the *tensorEVD* function (see Supplementary Note 3 for an actual numerical example).

**Table:** 

EVD = tensorEVD(K1, K2, ID1, ID2, alpha = 0.95) ncol(EVD$vectors) # Number of eigenvectors sum(EVD$values)/EVD$totalVar # Variance explained

## Results and discussion

We benchmarked the *tensorEVD* routine against the *eigen* function of the “base” R package (R Core Team 2021) in terms of the computational time used to derive eigenvectors, the accuracy of the approximation provided by *tensorEVD*, and the dimension of the resulting basis. All the analyses were performed in R v4.2.0 (R Core Team 2021) run on the High Performance Computing Center (HPCC) from Michigan State University (https://icer.msu.edu/hpcc/hardware) using nodes equipped with Intel Xeon Gold 6148 CPUs at 2.40 GHz with 84 GB of RAM memory in a single computing thread.

The data used in these benchmarks was generated by the Genomes to Fields (G2F) Initiative (Lima *et al.* 2023), which was curated and expanded by adding environmental covariates by Lopez-Cruz *et al.* (2023). This data set was used to derive a genetic (GRM) and an environmental relationship matrix (ERM, from the environmental covariates, see Supplementary Note 4) for 4,344 maize hybrids and 97 environments (year–locations), respectively, corresponding to the northern testing locations. We formed Hadamard products [K in Equation (2)] between the GRM (as $K_{1}$) and the ERM (as $K_{2}$) matrix of various sizes by sampling hybrids ($n_{G}=100,500,$ and 1,000), environments ($n_{E}=10,30,$ and 50), and the level of replication needed to complete a total sample size ranging from *n* = 10,000 to 30,000. Then, we factorized the resulting Hadamard product matrix using the R base function *eigen* (R Core Team 2021) as well as using *tensorEVD*, deriving as many eigenvectors as needed to explain 90, 95, and 98% of the total variance.

**Fig. 1.**
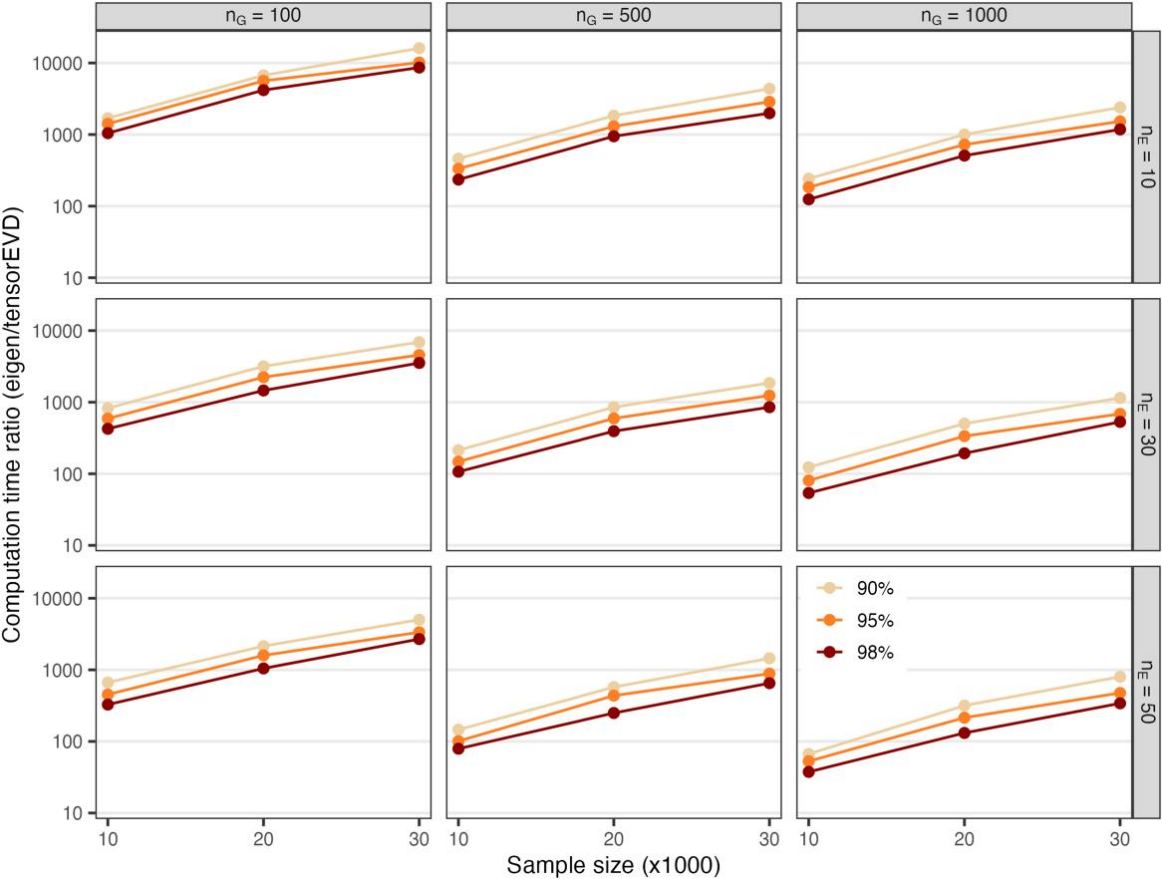
Computation time ratio (log_10_ scale, average across 20 replicates) of the EVD of the matrix **K** using the *eigen* method relative to *tensorEVD* method, by sample size (n=10,000,20,000, and 30,000 in the *x*-axis) and proportion *α* of variance of **K** explained (α=0.90,0.95, and 0.98). Each panel represents a combination of number of hybrids ($n_{G}$) and number of environments ($n_{E}$).

The *tensorEVD* method was consistently orders of magnitude faster than *eigen* (see Supplementary Figs. 1–3). The difference in computation time is particularly clear (e.g. *tensorEVD* ∼10,000 faster than *eigen*) when the product of the dimensions of each of the relationship matrices ($n_{G}\times n_{E})$ was smaller than sample size (*n*)—compare the left, middle, and right columns of Fig. 1.

The Cholesky decomposition is an alternative factorization that can be used to implement the models discussed in this study. This factorization has a computational complexity of $O\left(\frac{1}{3}n^{3}\right)$, which is smaller than the complexity of the EVD [$O(n^{3})$]. However, the Cholesky decomposition can be numerically unstable for matrices that are (near) singular, a situation that is not uncommon in G × E models. Another approach would be to use partial EVD methods that compute only a fraction of the eigenvectors. To evaluate these approaches, we benchmarked *tensorEVD* against the Cholesky decomposition as per the *chol* function from the “base” R package (R Core Team 2021) and against partial EVD computed using the *trlan.eigen* and *eigs_sym* functions from the “svd” (Korobeynikov *et al.* 2023) and “RSpectra” (Qiu and Mei 2022) R packages, respectively. As expected, partial SVD methods were faster than *eigen* only when the product $n_{G}\times n_{E}$ was smaller than *n* (see Supplementary Figs. 1–3); however, *tensorEVD* was much faster than the partial EVD methods (Supplementary Fig. 4). Likewise, *tensorEVD* was faster than *chol* only in cases where $n_{G}\times n_{E}<n$.

### Approximation accuracy

**Fig. 2.**
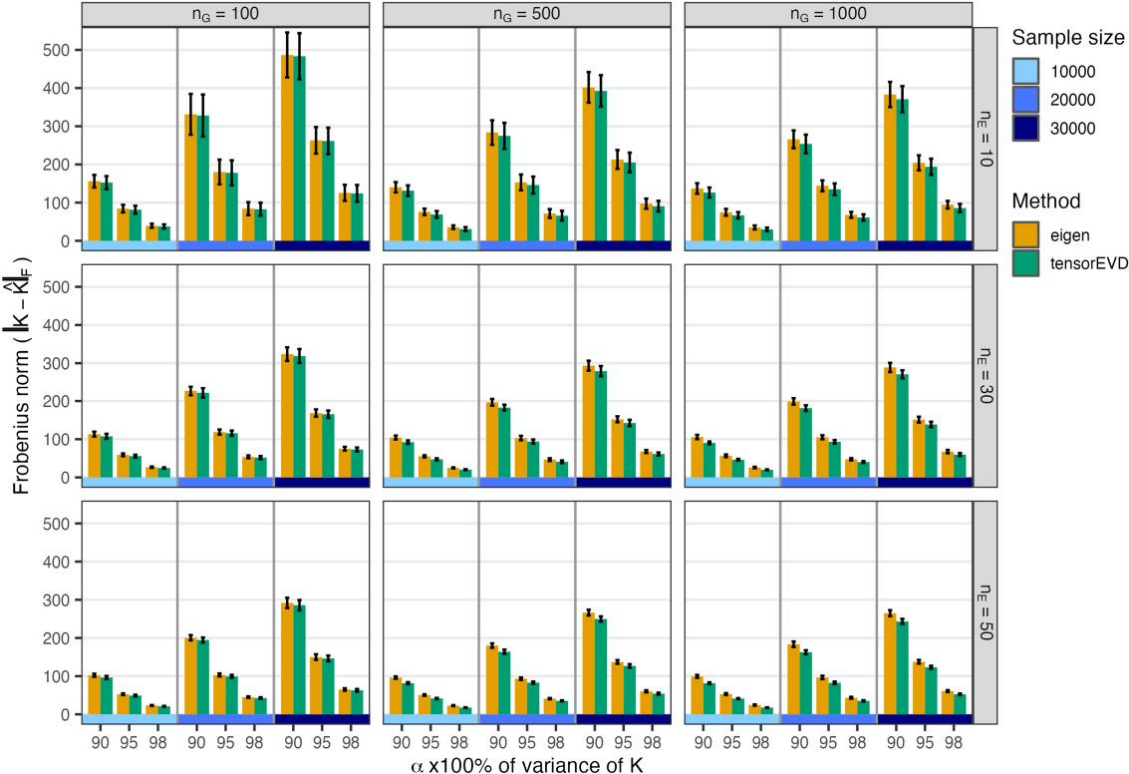
Frobenius norm (average ± SD across 20 replicates) of the difference between the Hadamard matrix **K** and the approximation (${\hat{K}}_{\alpha }$) provided by the *eigen* and *tensorEVD* procedures, by sample size (n=10,000,20,000, and 30,000) and proportion *α* of variance of **K** explained (α=0.90,0.95, and 0.98). Each panel represents a combination of number of hybrids ($n_{G}$) and number of environments ($n_{E}$). Smaller norm indicates better approximation.

We measured the accuracy of the approximation of the basis derived by the *eigen* and *tensorEVD* routines for each of the *α*-values by evaluating the Frobenius norm (i.e. a matrix generalization of the Euclidean norm; Golub and Van Loan 1996) of the difference between the Hadamard product matrix (**K**) and the approximation (${\hat{K}}_{\alpha }={\tilde{V}}_{\alpha }{\tilde{D}}_{\alpha }{\tilde{V}}_{\alpha }^{\prime }$, where ${\tilde{V}}_{\alpha }$ and ${\tilde{D}}_{\alpha }$ are the eigenvectors and eigenvalues derived by each method and *α*-value; see Supplementary Note 5 for more details). In general, both methods provided a very good and similar approximation (Fig. 2). As expected, the values of the norm decrease when *α* increased (smaller norm indicates better approximation). The values of the norm for different sample sizes cannot be compared because the Frobenius norm is a cumulative sum of n×n elements. Therefore, we also computed the correlation matrix distance (CMD; Herdin *et al.* 2005) between the Hadamard product matrix (**K**) and the approximation provided by each method and *α*-value (${\hat{K}}_{\alpha }$; see Supplementary Note 5 and Fig. 5). These CMD values are always between 0 and 1. In all the cases, the CMD was very small (<0.006), which indicates that both approximations were very good. As with the Frobenius norm metric, the CMD shows that both methods provide similar approximations (Supplementary Fig. 5); however, there is evidence that whenever $n_{G}\times n_{E}$ becomes larger than sample size *n*, *tensorEVD* provides a slightly better approximation than the *eigen* method (e.g. bottom-right panel in Fig. 2 and Supplementary Fig. 5, see Supplementary Fig. 6).

### Dimension reduction

**Fig. 3.**
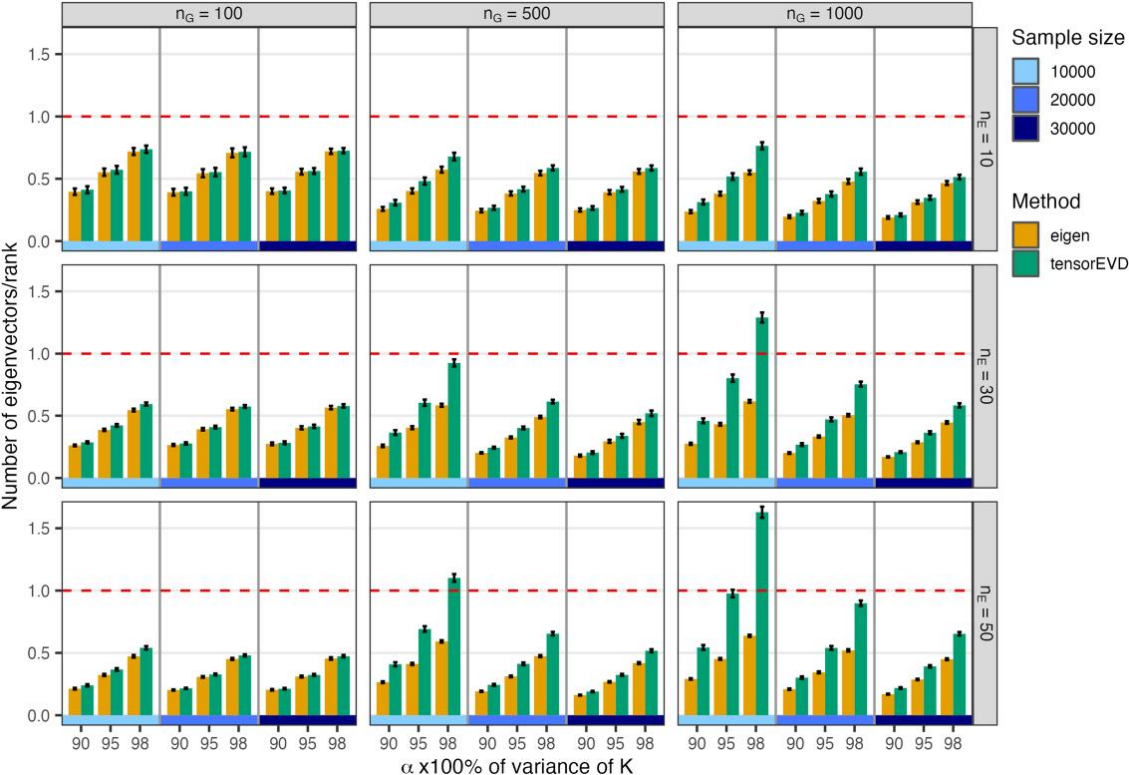
Number of eigenvectors (average ± SD across 20 replicates) produced by the *eigen* and *tensorEVD* methods, relative to the rank of matrix **K**, by *α*-value (i.e. proportion of variance explained, α=0.90,0.95, and 0.98) and sample size (n=10,000,20,000, and 30,000). Each panel represents a combination of number of hybrids ($n_{G}$) and number of environments ($n_{E}$).

We also compared *eigen* and *tensorEVD* in terms of the number of eigenvectors provided by each method for a given *α*-value, relative to the rank of the **K** (i.e. the number of eigenvectors of **K** with positive eigenvalue). By construction, *eigen* is very efficient at maximizing the proportion of variance explained in the derivation of eigenvectors. The *tensorEVD* function is as effective as the *eigen* method at dimension reduction only for cases where $n_{G}\times n_{E}<n$, for example the case $n_{G}=100$ and $n_{E}=10$ (top-left panel in Fig. 3). However, *tensorEVD* becomes less effective at dimension reduction when $n_{G}\times n_{E}$ exceeds sample size *n* (e.g. bottom-right panel in Fig. 3, see Supplementary Fig. 7).

### Application in genomic prediction

Finally, we evaluated the performance of the approximation of K provided by the *tensorEVD* method in Gaussian linear models in terms of variance component estimates and cross-validation prediction accuracies. For this evaluation, we used all the G2F data from the northern testing locations included in the data set presented by Lopez-Cruz *et al.* (2023). For the northern testing locations, this data set includes n=59,069 records for 4 traits (grain yield, anthesis, silking, and anthesis–silking interval) from $n_{G}=4,344$ hybrids and $n_{E}=97$ environments.

We analyzed these data with a Gaussian reaction norm model (Jarquín *et al.* 2014) in which the response ($y_{ijk}$) is modeled as the sum of the main effect of hybrid ($G_{i}$), main effect of environment ($E_{j}$), and the interaction hybrid × environment ($GE_{ij}$) term, and this is as follows:


(3) $$y_{ijk}=\mu +G_{i}+E_{j}+GE_{ij}+\varepsilon_{ijk}.$$

Above, *μ* is an intercept and *i*, *j*, and *k* are indices for the hybrids, environment, and replicate, respectively. The term $\varepsilon_{ijk}$ is an error term assumed to be Gaussian distributed as $\varepsilon_{ijk}\overset{iid}{∼}\;N(0,\sigma_{\varepsilon }^{2})$, with $\sigma_{\varepsilon }^{2}$ variance parameter associated to the error. Hybrid, environment, and interaction effects were assumed to be multivariate normally distributed with zero mean and effect-specific covariance matrices, specifically $G∼\mathrm{MVN}(0,\sigma_{G}^{2}K_{G})$, $E∼\mathrm{MVN}(0,\sigma_{E}^{2}K_{E})$, and $GE∼\mathrm{MVN}(0,\sigma_{\mathrm{GE}}^{2}K)$, where K takes the Hadamard form in Equation (2), $K=(Z_{1}K_{G}Z_{1}^{\prime })\circ (Z_{2}K_{E}Z_{2}^{\prime })$, and $\sigma_{G}^{2}$, $\sigma_{E}^{2}$, and $\sigma_{\mathrm{GE}}^{2}$ are variance parameters associated to G, E, and GE, respectively. We fitted the model in Equation (3) to each trait in a Bayesian fashion using the “BGLR” R package (Pérez-Rodríguez and de los Campos 2022) with the decomposition of the GE kernel (K) computed using *eigen* and *tensorEVD* methods for different percentages of variance of K explained (α=0.90,0.95, and 0.98). For these analyses, we used computing nodes equipped with Intel Xeon E5-2680 v4 CPUs at 2.40 GHz with 96 GB of RAM memory using 3 computing threads.

**Fig. 4.**
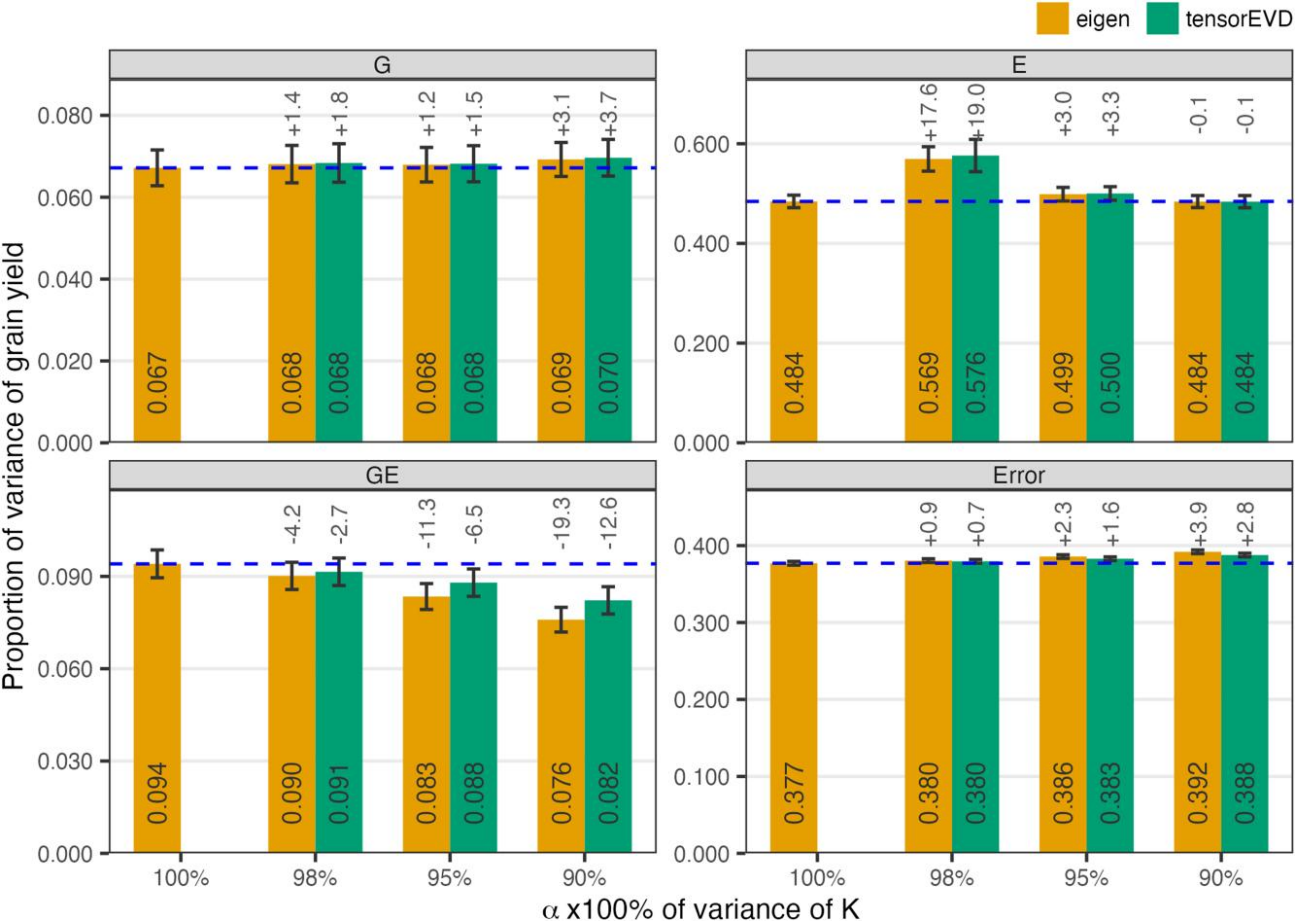
Proportion of the phenotypic variance (average ± SD across 5 replicates) of grain yield explained by each model term (G, E, GE, and Error) in Equation (3). The EVD of the Hadamard matrix **K** (covariance matrix of GE) was performed using *eigen* and *tensorEVD* methods for different *α*-values (α=1.00,0.98,0.95,and0.90). Numbers on the top represent the percentage of change (%) relative to the variance explained by each term in the model that uses full information in **K** (i.e. α=1.00) obtained with the *eigen* method (horizontal dotted line).

As one would expect, reducing *α* from 1 to 0.98, 0.95, and 0.90 led to a slight reduction in the proportion of variance explained by the GE term and a small increase in the error variance (Fig. 4). In general, for the same *α*-value, the reduction in proportion of variance explained and the increase in the error variance was smaller with the *tensorEVD* compared to *eigen*. We obtained similar patterns for anthesis, silking, and anthesis–silking interval (Supplementary Figs. 8–10).

**Fig. 5.**
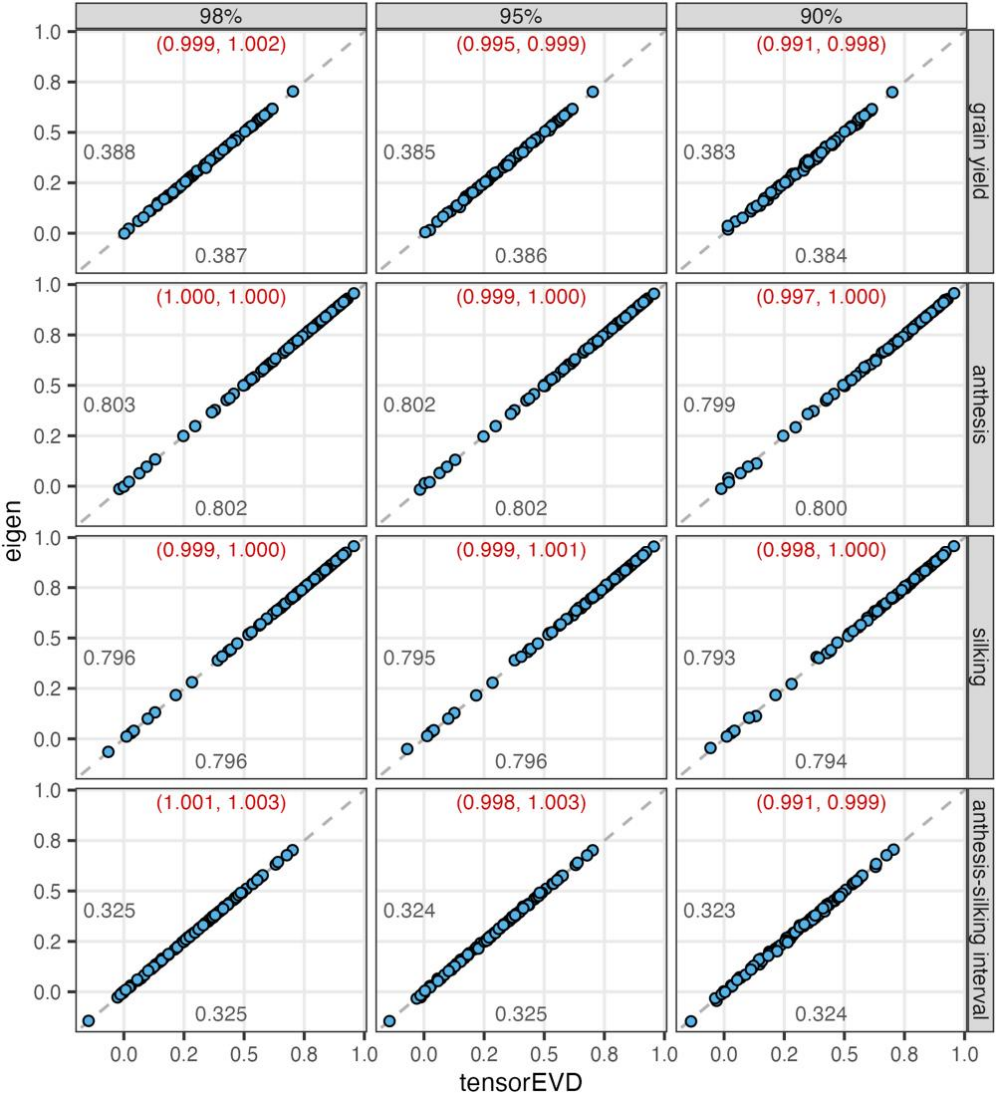
Within environment prediction correlation (*r*) for the model in Equation (3) in cross-validation with EVD of the Hadamard matrix **K** performed using the *tensorEVD* (*x*-axis) and the *eigen* (*y*-axis) method. Each point gives the prediction correlation obtained within an environment ($r_{i}$, i=1,…,97) with each of the methods, by trait (rows) and *α*-value (in columns, proportion of variance of **K** explained captured by the selected eigenvectors). Numbers (below and above the diagonal) represent the weighted mean across the 97 environments for each of the methods. The numbers in parenthesis are 95% C.I. for the coefficient *b* in the regression $r_{\mathrm{eigen}}=a+b\;r_{\mathrm{tensorEVD}}+\varepsilon$.

To evaluate *prediction performance*, we conducted a 10-fold cross-validation with hybrids assigned to folds (this mimics the CV1 scheme of Burgueño *et al.* 2012). For any given *α*-value, the models fitted using the factorization derived with *tensorEVD* and *eigen* produced almost identical predictions (Fig. 5). Furthermore, there was a negligible reduction in prediction accuracy associated to lower values of *α*. For instance, for grain yield, the prediction correlations with the *tensorEVD* method were 0.387, 0.386, and 0.384 for *α*-values of 0.98, 0.95, and 0.90, respectively (Fig. 5).

In the analysis presented above, the Hadamard product matrix had a dimension of n=59,059 and a rank (number of eigenvalues greater than 0) of 38,187. Table 1 gives the number of eigenvectors returned by *eigen* and *tensorEVD* by *α*-value. As previously noted, *tensorEVD* is less efficient than *eigen* at dimension reduction when $n_{G}\times n_{E}\gg n$, a condition met in the analysis just described (4,344×97≫59,059). However, using an α=0.95  *tensorEVD* already provides substantial dimension reduction that translates into a shorter total computation time (decomposition + model fitting, Table 1; Supplementary Fig. 11).

**Table 1.** Number of eigenvectors and the times required to perform EVD and to generate 50,000 posterior samples by method and target proportion of variance explained.

α× 100% of variance	Method	Number of eigenvectors	Time to compute the EVD (min)*^a^*	Time in the Gibbs sampling*^b^*		Total time (min)*^e^*
				Per sample (sec)*^c^*	Per 50,000 samples (min)*^d^*	
100%	eigen	38,187	267.56 (2.82)	0.670 (0.058)	576.61 (38.99)	844.17
98%	eigen	12,294	266.39 (3.10)	0.111 (0.003)	98.64 (2.36)	365.02
	tensorEVD	37,442	3.13 (0.17)	0.631 (0.023)	545.51 (18.38)	548.65
95%	eigen	7,260	266.01 (3.55)	0.062 (0.002)	56.60 (1.08)	322.61
	tensorEVD	19,843	2.78 (0.17)	0.217 (0.007)	190.53 (5.87)	193.31
90%	eigen	3,839	267.66 (2.76)	0.035 (0.001)	33.18 (0.68)	300.85
	tensorEVD	9,512	2.39 (0.11)	0.080 (0.002)	72.31 (1.12)	74.70

^*a*^Average (SD) across 10 replicates of the decomposition.

^*b*^Gibbs sampler was implemented using BGLR for the model in Equation (3) fitted to each trait (grain yield, anthesis, silking, and anthesis–silking interval). Each model was run with 50,000 MCMC iterations (discarding 5,000 as burning and using a thinning of 10 samples) and replicated 5 times.

^*c*^Average (SD), across 4 traits and 5 replicates, time per iteration (median value across iterations).

^*d*^Average (SD), across 4 traits and 5 replicates, time to perform the Gibbs sampler, which includes the initial overheading time (matrix preparation and hyperparameter setting) plus time to complete all iterations.

^*e*^Estimated total computing time (EVD computation + Gibbs sampling). All the computations were carried out on the MSU's HPCC in nodes with Intel processors with 96 GB of RAM memory using 3 computing threads.

## Conclusion

The *tensorEVD* method can be used to factorize large Hadamard product matrices that are submatrices of Kronecker products of smaller positive semidefinite matrices. Examples where such matrices are key components of genomic models include hybrid prediction and G × E models involving genetic and environmental relationship matrices. The proposed algorithm can be several orders of magnitude faster than a standard EVD, with relatively negligible effect on variance component estimates and prediction performance. The proposed method can be very advantageous in terms of speed and dimensionality reduction in cases where the dimensions of the low-rank matrices are very small relative to the sample size.

## Web resources

The “tensorEVD” R package is freely available on CRAN (https://CRAN.R-project.org/package=tensorEVD) and on the GitHub repository (https://github.com/MarcooLopez/tensorEVD). All the scripts used for analyses can be found in the “tensorEVD” R package documentation.

## Supplementary Material

jkae001_Supplementary_Data

## Data Availability

The experimental field trial and genomic data sets used in this study for the simulation and genomic prediction applications were generated by the Genomes to Fields (G2F) Initiative. The curated and expanded data set that includes the environmental covariates was obtained from the study of Lopez-Cruz *et al.* (2023), available in the Figshare repository (https://doi.org/10.6084/m9.figshare.22776806). All Supplementary Notes 1–5 and Supplementary Figs. 1–11 are included in the Supplementary material file, which is provided along with this manuscript. Supplemental material available at G3 online.
