# Supplementary material for: A fast algorithm to factorize high-dimensional tensor product matrices used in genetic models
Source: G3 (Bethesda). 2024 Jan 5;14(3):jkae001. doi: 10.1093/g3journal/jkae001 (PMC11090460; doi:10.1093/g3journal/jkae001)
Supplement: jkae001_Supplementary_Data [file jkae001_supplementary_data.docx]

#

# Supplementary Material

A fast algorithm to factorize high-dimensional

Tensor Product matrices used in Genetic Models

Marco Lopez-Cruz*, Paulino Pérez-Rodríguez & Gustavo de los Campos

* Corresponding author: MLC ([lopezcru@msu.edu](mailto:lopezcru@msu.edu))

This file contains:

- Supplementary Notes 1-5
- Supplementary Figures 1-11

**Supplementary Notes**

**Supplementary Note 1**

Consider the Kronecker product of two symmetric positive semi-definite matrices, $\mathbf{K}_{1}$ and $\mathbf{K}_{2}$,

$$\mathbf{K}=\mathbf{K}_{1}\bigotimes\mathbf{K}_{2}.$$

Let the eigenvalue decomposition (EVD) of the matrices $\mathbf{K}_{1}$ and $\mathbf{K}_{2}$ in the right-hand side be $\mathbf{K}_{1}=\mathbf{V}_{1}\mathbf{D}_{1}\mathbf{V}_{1}^{\boldsymbol{'}}$ and $\mathbf{K}_{2}=\mathbf{V}_{2}\mathbf{D}_{2}\mathbf{V}_{2}^{\boldsymbol{'}}$, respectively. Replacing these matrices with their EVD we get:

$\mathbf{K}=\left( \mathbf{V}_{1}\mathbf{D}_{1}\mathbf{V}_{1}^{\boldsymbol{'}} \right)\bigotimes\left( \mathbf{V}_{2}\mathbf{D}_{2}\mathbf{V}_{2}^{\boldsymbol{'}} \right).$ (1)

Assume the EVD of the Kronecker to be $\mathbf{K}=\mathbf{VD}\mathbf{V}^{'}$. Below, we show that the eigenvectors ($\mathbf{V}$) and eigenvalues (the diagonal entries of $\mathbf{D}$**)** of **K** are Kronecker products of the eigenvectors and eigenvalues of $\mathbf{K}_{1}$ and $\mathbf{K}_{2}$, i.e.,

$\mathbf{VD}\mathbf{V}^{'}=\left( \mathbf{V}_{1}\bigotimes\mathbf{V}_{2} \right)\left( \mathbf{D}_{1}\bigotimes\mathbf{D}_{2} \right)\left( \mathbf{V}_{1}\bigotimes\mathbf{V}_{2} \right)^{'}.$ (2)

*Proof*:

Recall the following property of Kronecker products (e.g., Searle 1982, p. 265):

$\left( \mathbf{AB} \right)\bigotimes\left( \mathbf{CD} \right)=\left( \mathbf{A}\bigotimes\mathbf{C} \right)\left( \mathbf{B}\bigotimes\mathbf{D} \right)$.

Applying this property recursively to the right-hand side of Eq. (1), we get:

$$\left[ \left( \mathbf{V}_{1} \right)\left( \mathbf{D}_{1}\mathbf{V}_{1}^{\boldsymbol{'}} \right) \right]\bigotimes\left[ \left( \mathbf{V}_{2} \right)\left( \mathbf{D}_{2}\mathbf{V}_{2}^{\boldsymbol{'}} \right) \right]=\left[ \left( \mathbf{V}_{1}\bigotimes\mathbf{V}_{2} \right) \right]\left[ \left( \mathbf{D}_{1}\mathbf{V}_{1}^{\boldsymbol{'}} \right)\bigotimes\left( \mathbf{D}_{2}\mathbf{V}_{2}^{\boldsymbol{'}} \right) \right]$$

$$=\left[ \left( \mathbf{V}_{1}\bigotimes\mathbf{V}_{2} \right) \right]\left[ \left( \mathbf{D}_{1}\bigotimes\mathbf{D}_{2} \right)\bigotimes\left( \mathbf{V}_{1}^{\boldsymbol{'}}\bigotimes\mathbf{V}_{2}^{\boldsymbol{'}} \right) \right]$$

$$=\left( \mathbf{V}_{1}\bigotimes\mathbf{V}_{2} \right)\left( \mathbf{D}_{1}\bigotimes\mathbf{D}_{2} \right)\left( \mathbf{V}_{1}\bigotimes\mathbf{V}_{2} \right)^{'}$$

where the last equality comes from the property $\left( \mathbf{A}\bigotimes\mathbf{B} \right)^{'}=\mathbf{A}^{'}\bigotimes\mathbf{B}^{'}$. Furthermore,

- The term $\mathbf{V}=\mathbf{V}_{1}\bigotimes\mathbf{V}_{2}$ can be shown to be orthonormal, i.e., $\mathbf{V}'\mathbf{V}=\mathbf{I}$, and
- The term $\mathbf{D}=\mathbf{D}_{1}\bigotimes\mathbf{D}_{2}$ can be shown to be diagonal.

Therefore, $\mathbf{V}$ and the diagonal elements of $\mathbf{D}$ are the eigenvectors and eigenvalues of $\mathbf{K}$, respectively (Eq. (2)).

**Supplementary Note 2**

The snippet below shows a numerical toy example of the result in Eq. (2).

| set.seed(195021)  # Simulate matrices K1 and K2 of dimension n1 and n2  n1 = 4; n2 = 5  K1 = crossprod(matrix(rnorm(n1*n1), ncol=n1))  K2 = crossprod(matrix(rnorm(n2*n2), ncol=n2))  # Direct EVD of the Kronecker product  K = kronecker(K1,K2)  EVD = eigen(K)  V0 = EVD$vectors # eigenvectors in left-hand side Eq.(2)  D0 = EVD$values # eigenvalues in left-hand side Eq.(2)  # Tensor EVD using K1 and K2  EVD1 = eigen(K1)  EVD2 = eigen(K2)  V = kronecker(EVD1$vectors, EVD2$vectors) # eigenvectors in right-hand side Eq.(2)  D = kronecker(EVD1$values, EVD2$values) # eigenvalues in right-hand side in Eq.(2) |
| --- |

Eigenvalues $\mathbf{D}_{1}\bigotimes\mathbf{D}_{2}$ in the right-hand side of Eq. (2) are not necessary sorted from largest to smallest, hence we have to sort them, eigenvectors $\mathbf{V}_{1}\bigotimes\mathbf{V}_{2}$ are sorted accordingly as well

| index = order(D, decreasing=TRUE)  D = D[index]  V = V[,index] |
| --- |

After sorting, the maximum difference in eigenvalues ($\mathbf{D}$ vs $\mathbf{D}_{1}\bigotimes\mathbf{D}_{2}$) is approximate zero

| max(abs(D-D0))  # [1] 6.82121e-13 |
| --- |

The eigenvectors ($\mathbf{V}$ and $\mathbf{V}_{1}\bigotimes\mathbf{V}_{2}$) are also identical up to a factor $k$ equal to 1 or -1.

| dat = dat2 = c()  for(j in 1:ncol(V)){  dat = rbind(dat,data.frame(v=paste("Eigenvector",j),x=V[,j],y=V0[,j]))  dat2 = rbind(dat2,data.frame(v=paste("Eigenvector",j),a=cor(V[,j],V0[,j])))  }  dat$v = factor(dat$v, levels=paste("Eigenvector",seq(ncol(V))))  dat2$v = factor(dat2$v, levels=levels(dat$v))  library(ggplot2)  ggplot(dat, aes(x,y)) + theme_bw() +  geom_abline(data=dat2, aes(intercept=0, slope=a), color="gray50") +  geom_point(shape=1, color="red3") + facet_wrap(~v) +  labs(x=bquote("Kronecker("*V[1]*","*V[2]*")"), y='V') |
| --- |

**Supplementary Note 3**

Code below shows how to perform EVD using the *tensorEVD* function. First, matrices $\mathbf{K}_{1}$and $\mathbf{K}_{2}$ of dimensions $n_{1}$ and $n_{2}$, respectively, will be simulated. Then, integer $n$-vectors ${ID}_{1}$ and ${ID}_{2}$ will be simulated to form the Hadamard product. Finally, the eigenvectors and eigenvalues provided by *tensorEVD* will be compared with those obtained with the *eigen* R-function.

| library(tensorEVD)  set.seed(195021)  n1 = 20; n2 = 30  K1 = crossprod(matrix(rnorm(n1*(n1+10)), ncol=n1))  K2 = crossprod(matrix(rnorm(n2*(n2+10)), ncol=n2)) |
| --- |

**Example 1**. Full design (Kronecker product).

For a full design (i.e., exactly $n_{1}\times n_{2}$ combinations, each appearing only once), *tensorEVD* and *eigen* produce the same results (as shown in Supplementary Note 2).

| ID1 = rep(seq(n1), each=n2)  ID2 = rep(seq(n2), times=n1)  # Direct EVD of the Hadamard product  K = K1[ID1,ID1]*K2[ID2,ID2]  EVD0 = eigen(K)  # Tensor EVD using K1 and K2  EVD = tensorEVD(K1, K2, ID1, ID2)  # Eigenvectors and eigenvalues are numerically equal  max(abs(EVD$values-EVD0$values))  # [1] 2.728484e-11  max(abs(EVD$vectors)-abs(EVD0$vectors))  # [1] 6.628337e-12 |
| --- |

If a proportion of variance explained ($0<\alpha\leq1$, e.g., $\alpha=0.95$) is specified, only the eigenvectors needed to explain such proportion are derived

| alpha = 0.95  EVD = tensorEVD(K1, K2, ID1, ID2, alpha=alpha)  dim(EVD$vectors)  # [1] 600 348  # For the direct EVD  varexp = cumsum(EVD0$values/sum(EVD0$values))  index = 1:which.min(abs(varexp-alpha))  V = EVD0$vectors[,index]  dim(V)  # [1] 600 348 |
| --- |

**Example 2**. Incomplete design (Hadamard product).

For incomplete designs, eigenvectors and eigenvalues are no longer equivalent

| n = n1*n2 # Sample size n  ID1 = sample(seq(n1), n, replace=TRUE) # Randomly sample of ID1  ID2 = sample(seq(n2), n, replace=TRUE) # Randomly sample of ID2  K = K1[ID1,ID1]*K2[ID2,ID2]  EVD0 = eigen(K)  EVD = tensorEVD(K1, K2, ID1, ID2)  max(abs(EVD$values-EVD0$values))  # [1] 2725.911  max(abs(EVD$vectors)-abs(EVD0$vectors))  # [1] 0.6775934 |
| --- |

However, the sum of the eigenvalues in both methods is equal to the $trace(\mathbf{K})$ (i.e., the sum of the diagonal elements of $\mathbf{K}$) and provide the same approximation for $\mathbf{K}=\mathbf{VD}\mathbf{V}^{'}$

| c(sum(EVD0$values), sum(EVD$values), sum(diag(K)))  # [1] 710693.3 710693.3 710693.3  # And provide the same approximation for K  K01 = EVD0$vectors%*%diag(EVD0$values)%*%t(EVD0$vectors)  K02 = EVD$vectors%*%diag(EVD$values)%*%t(EVD$vectors)  c(max(K-K01), max(K-K02))  # [1] 1.136868e-11 1.045919e-11 |
| --- |

Note that when $n$ is different than the product $n_{1}\times n_{2}$, both methods provide different number or eigenvectors/ eigenvalues. The *eigen* function provides an EVD where the number of eigenvectors is equal to the minimum between $n$ and $n_{1}\times n_{2}$; for the *tensorEVD* function, this number is always equal to $n_{1}\times n_{2}$.

| # Sample size n being half of n1 x n2  n = n1*n2/2  ID1 = sample(seq(n1), n, replace=TRUE)  ID2 = sample(seq(n2), n, replace=TRUE)  K = K1[ID1,ID1]*K2[ID2,ID2]  EVD0 = eigen(K)  EVD = tensorEVD(K1, K2, ID1, ID2)  c(ncol(EVD0$vectors), ncol(EVD$vectors))  # [1] 300 600  # Sample size n being twice n1 x n2  n = n1*n2*2  ID1 = sample(seq(n1), n, replace=TRUE)  ID2 = sample(seq(n2), n, replace=TRUE)  K = K1[ID1,ID1]*K2[ID2,ID2]  EVD0 = eigen(K)  EVD = tensorEVD(K1, K2, ID1, ID2)  c(ncol(EVD$vectors), ncol(EVD0$vectors))  # [1] 1200 600 |
| --- |

**Supplementary Note 4**

The data set was generated by the Genomes-To-Fields (G2F) initiative (Lima *et al.* 2023) which includes a genotypic data matrix for 4,372 maize hybrids and 98,026 single nucleotide polymorphisms (SNP). The data set also comprises an environmental covariates (EC) matrix derived by Lopez-Cruz *et al.* (2023) for 136 environments (year-locations) and 189 ECs.

The genetic relationship matrix (GRM, VanRaden 2008) was obtained as $\mathbf{K}_{G}=\mathbf{X}\mathbf{X}^{'}/\mathrm{trace}\left( \mathbf{X}\mathbf{X}^{'} \right)$, where $\mathbf{X}$ is the matrix of centered SNPs (hybrids in rows, SNPs in columns). Likewise, an environmental relationship matrix (ERM) was computed from the ECs as $\mathbf{K}_{E}=\mathbf{W}\mathbf{W}^{'}/\mathrm{trace}\left( \mathbf{W}\mathbf{W}^{\boldsymbol{'}} \right)$, where $\mathbf{W}$ is the matrix of centered and scaled ECs (environments in rows, ECs in columns). The GRM and ERM were calculated for the $4,344$ maize hybrids and $97$ environments, respectively, corresponding to the northern locations.

**Supplementary Note 5**

***Low-rank approximation from the EVD*.** Recall the eigenvalue decomposition (EVD) of an $N\times N$ positive semi-definite matrix $\mathbf{K}$ whose form is

$\mathbf{K}=\mathbf{VD}\mathbf{V}^{'}=\sum_{k=1}^{N} d_{k}\boldsymbol{v}_{k}\boldsymbol{v}_{k}^{\boldsymbol{'}}$ (3)

where $\mathbf{V=}\left[ \boldsymbol{v}_{1},...,\boldsymbol{v}_{N} \right]$ is an orthonormal matrix (i.e., $\mathbf{V}'\mathbf{V}=\mathbf{I}$) whose columns $\boldsymbol{v}_{k}$ ($k=1,\ldots,N$) are the eigenvectors and $\mathbf{D}=diag\left( d_{1},...,d_{N} \right)$ is a diagonal matrix with the eigenvalues $d_{1}\geq\ldots\geq d_{N}\geq0$.

It holds that the sum of the eigenvalues equals to the sum of the diagonal values of $\mathbf{K}$ (i.e., $trace(\mathbf{K})$), this is

$\sum_{k=1}^{N} d_{k}=trace(\mathbf{K})$.

Therefore, for $\mathbf{K}$ being a variance structure matrix, we say that the term $d_{k}/\sum_{k=1}^{N} d_{j}$ is the proportion of the total variability of $\mathbf{K}$ associated to the $k^{\mathrm{th}}$ eigenvector.

A low-rank approximation ($\hat{\mathbf{K}}$) of $\mathbf{K}$ can be formed by retaining the top $m$ eigenvectors (with $m<N$), those that jointly explain a proportion $\alpha$ of the total variance ($0<\alpha\leq1$, e.g., $\alpha=0.95$). This approximation is obtained by summing over the first $m$ terms in Eq. (3), this is ${\hat{\mathbf{K}}}_{\alpha}=\sum_{k=1}^{m} d_{k}\boldsymbol{v}_{k}\boldsymbol{v}_{k}^{\boldsymbol{'}}$, This is also equivalent to

$${\hat{\mathbf{K}}}_{\alpha} ={\tilde{\mathbf{V}}}_{\alpha}{\tilde{\mathbf{D}}}_{\alpha}{\tilde{\mathbf{V}}}_{\alpha}^{\mathbf{'}}$$

where ${\tilde{\mathbf{V}}}_{\alpha}\mathbf{=}\left[ \boldsymbol{v}_{1},...,\boldsymbol{v}_{m} \right]$ and ${\tilde{\mathbf{D}}}_{\alpha}=diag\left( d_{1},...,d_{m} \right)$ are matrices formed with the top $m$ eigenvectors and eigenvalues in $\mathbf{V}$ and $\mathbf{D}$, respectively, explaining a proportion $\alpha$ of the variance.

***Approximation accuracy*.** We measured the approximation error by calculating two metrics:

- Frobenius norm $\left\| \mathbf{A} \right\|_{F}$ (Golub and Van Loan 1996) of the matrix $\mathbf{A=K}-{\hat{\mathbf{K}}}_{\alpha}$, which is defined as the squared root of the sum of the squares of all the entries, i.e., $\left\| \mathbf{A} \right\|_{F}=\left( \sum_{i}^{N} \sum_{j}^{N} a_{ij}^{2} \right)^{1/2}$, where $a_{ij}$ is the entry in the $i^{\mathrm{th}}$ row and $j^{\mathrm{th}}$ column of $\mathbf{A}$.
- Correlation Matrix Distance $d(\mathbf{K}, {\hat{\mathbf{K}}}_{\alpha})$ (CMD, Herdin *et al.* 2005). This is defined as

$$d\left( \mathbf{A},\mathbf{B} \right)=1-\frac{trace(\mathbf{AB})}{\left\| \mathbf{A} \right\|_{F} \left\| \mathbf{B} \right\|_{F}}$$

which ranges between 0 and 1.

Note: Frobenius norm and DMC were calculated after transforming the covariance matrices $\mathbf{K}$ and ${\hat{\mathbf{K}}}_{\alpha}$ into correlation matrices.

**Supplementary Figures**


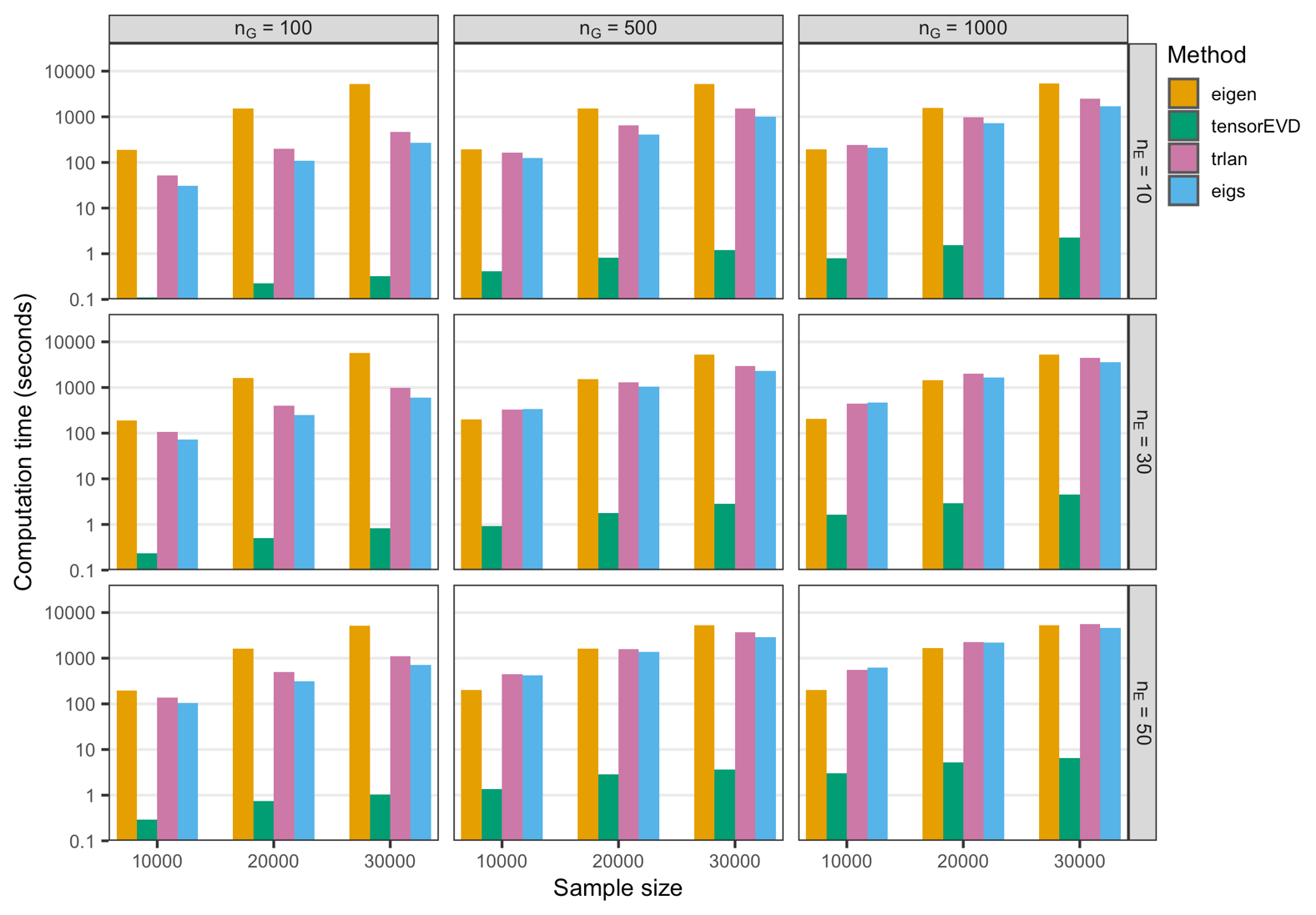


**Supplementary Figure 1.** Computing times (log_10_ scale, average across 20 replicates) of the EVD of the Hadamard matrix $\mathbf{K}$ using the *eigen*, *tensorEVD*, *trlan.eigen*, and *eigs_sym* methods, for a proportion $\alpha=0.90$ of variance of $\mathbf{K}$ explained, by sample size ($n=10,000, 20,000,$ and $30,000$ in the x-axis). Each panel represents a combination of number of hybrids ($n_{G}$) and number of environments ($n_{E}$).


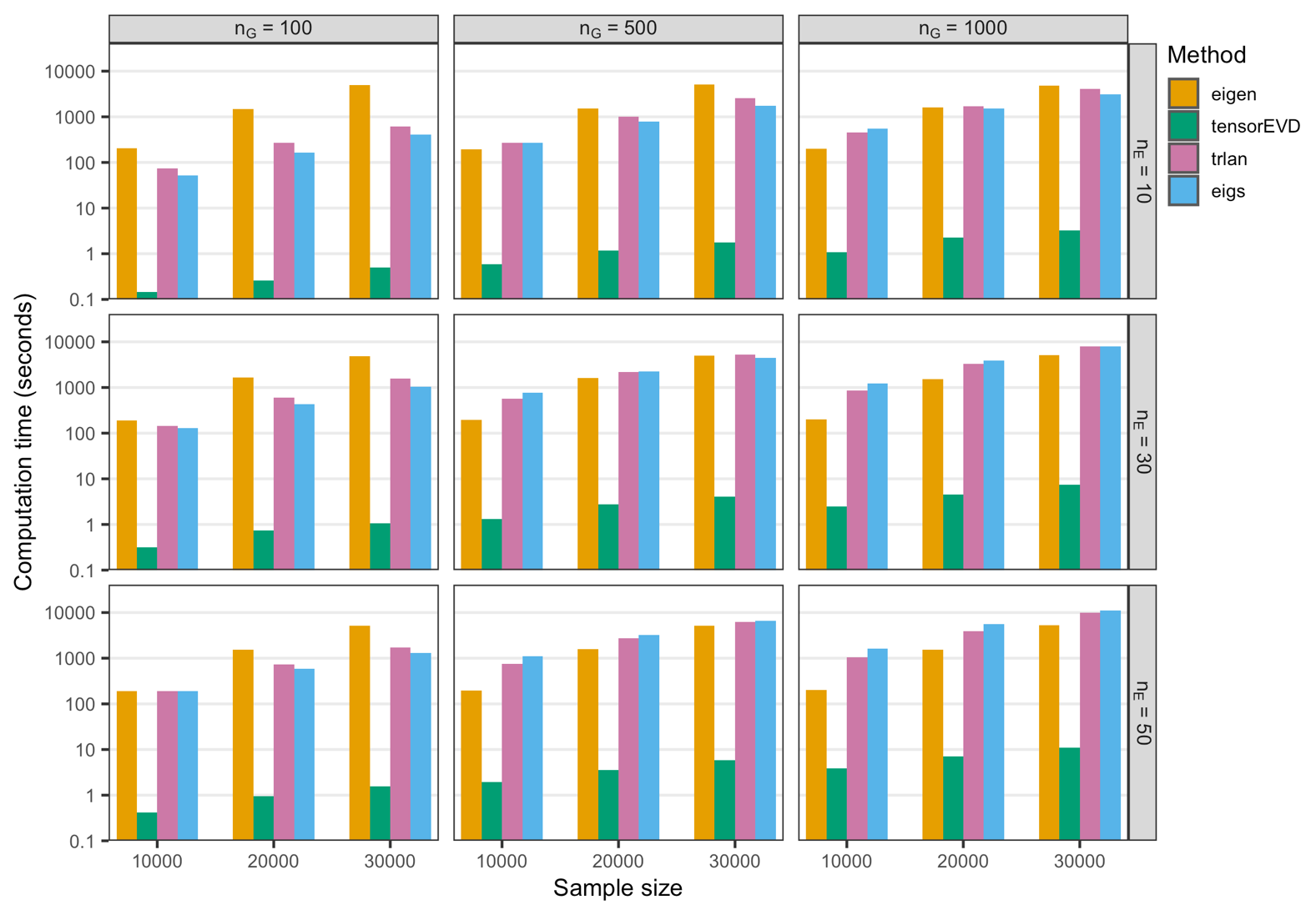


**Supplementary Figure 2.** Computing times (log_10_ scale, average across 20 replicates) of the EVD of the Hadamard matrix $\mathbf{K}$ using the *eigen*, *tensorEVD*, *trlan.eigen*, and *eigs_sym* methods, for a proportion $\alpha=0.95$ of variance of $\mathbf{K}$ explained, by sample size ($n=10,000, 20,000,$ and $30,000$ in the x-axis). Each panel represents a combination of number of hybrids ($n_{G}$) and number of environments ($n_{E}$).


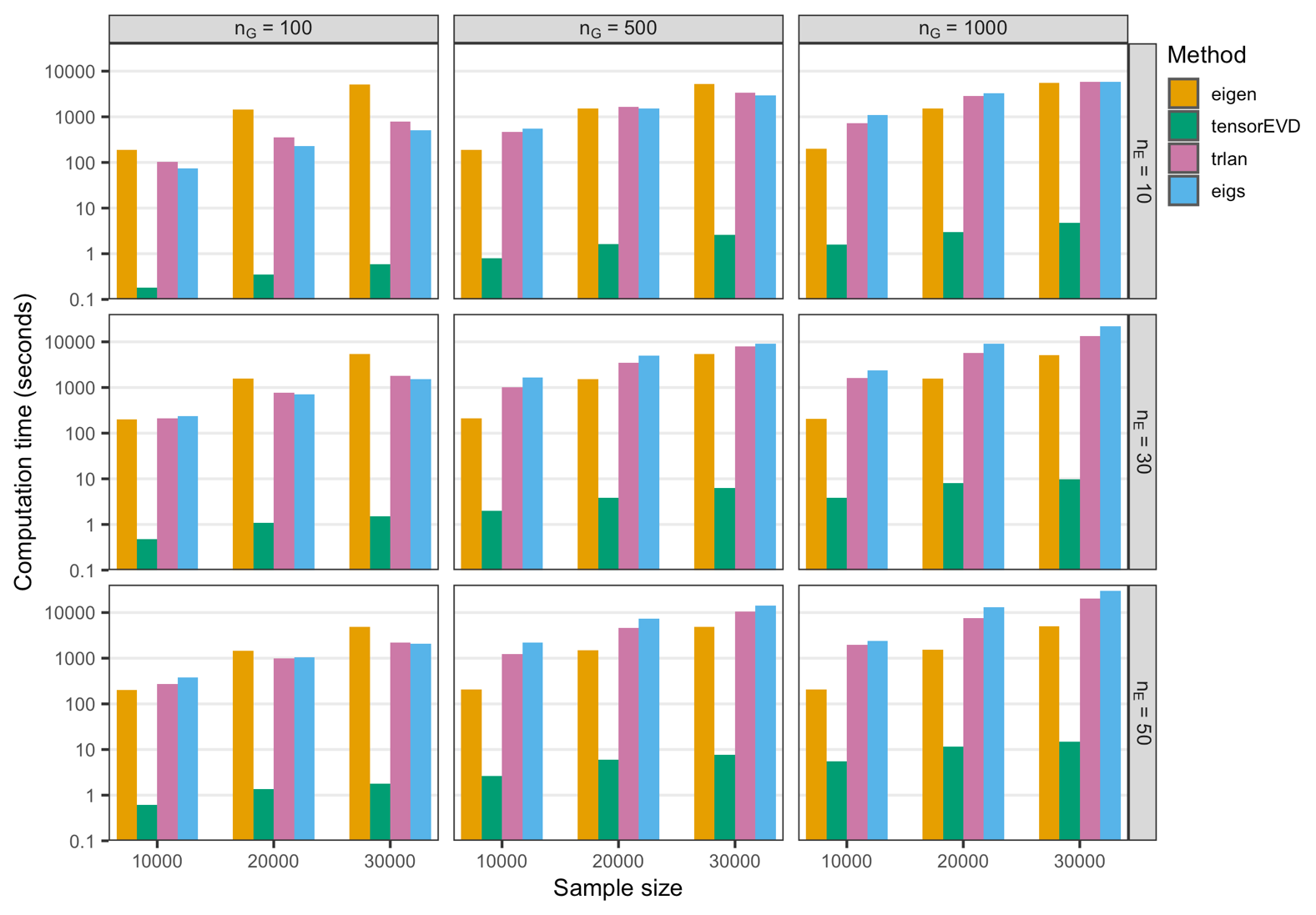


**Supplementary Figure 3.** Computing times (log_10_ scale, average across 20 replicates) of the EVD of the Hadamard matrix $\mathbf{K}$ using the *eigen*, *tensorEVD*, *trlan.eigen*, and *eigs_sym* methods, for a proportion $\alpha=0.98$ of variance of $\mathbf{K}$ explained, by sample size ($n=10,000, 20,000,$ and $30,000$ in the x-axis). Each panel represents a combination of number of hybrids ($n_{G}$) and number of environments ($n_{E}$).


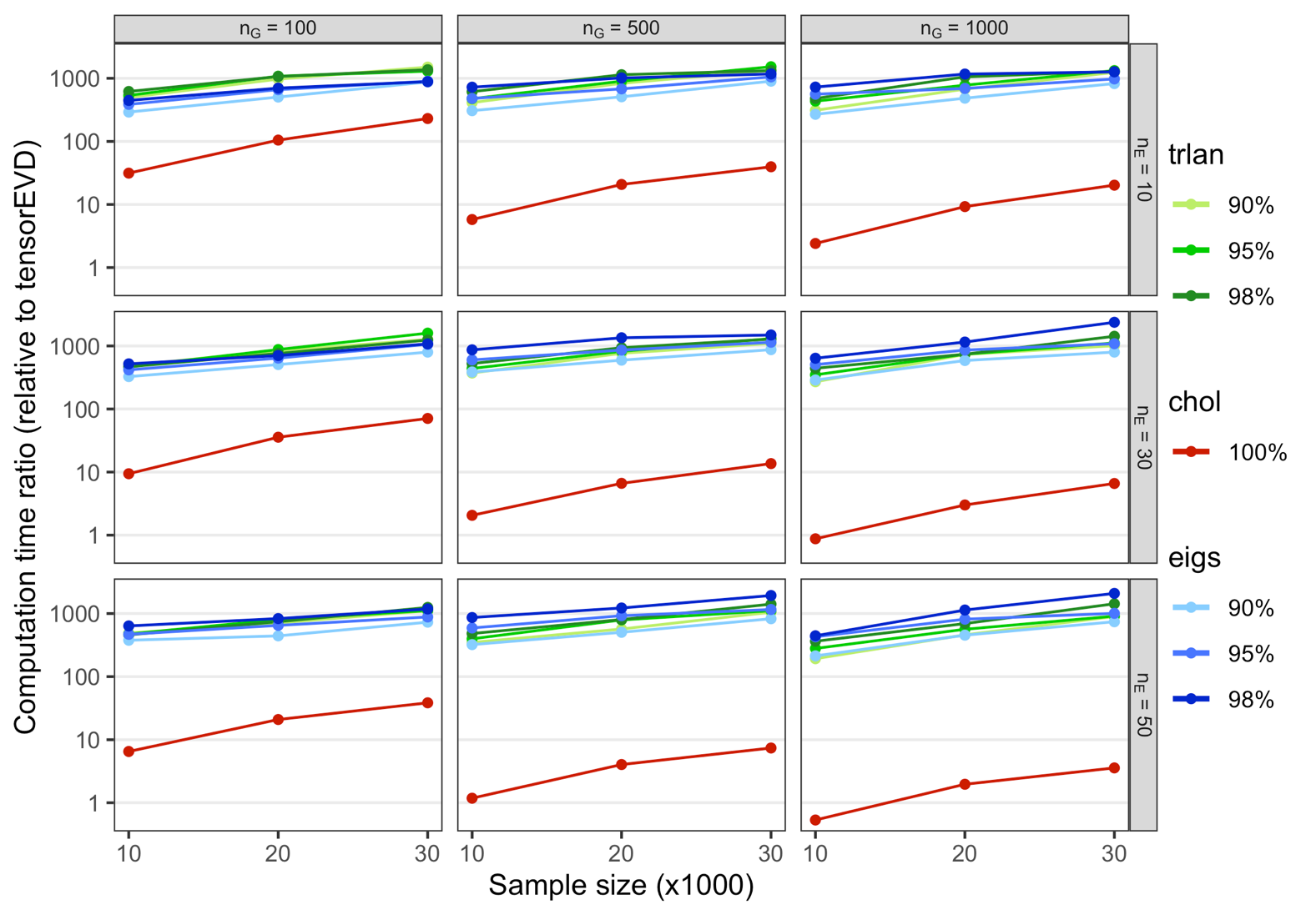


**Supplementary Figure 4.** Computation time ratio (log_10_ scale, average across 20 replicates) of the EVD of the matrix **K** using the *trlan.eigen*, *eigs_sym*, and *chol* methods, relative to *tensorEVD* method, by sample size ($n=10,000, 20,000,$ and $30,000$ in the x-axis) and proportion $\alpha$ of variance of **K** explained ($\alpha=0.90, 0.95,$ and $0.98$). Method *chol* does not perform dimensionality reduction, and therefore, it was compared with the *tensorEVD* at $\alpha=1.00$. Each panel represents a combination of number of hybrids ($n_{G}$) and number of environments ($n_{E}$).


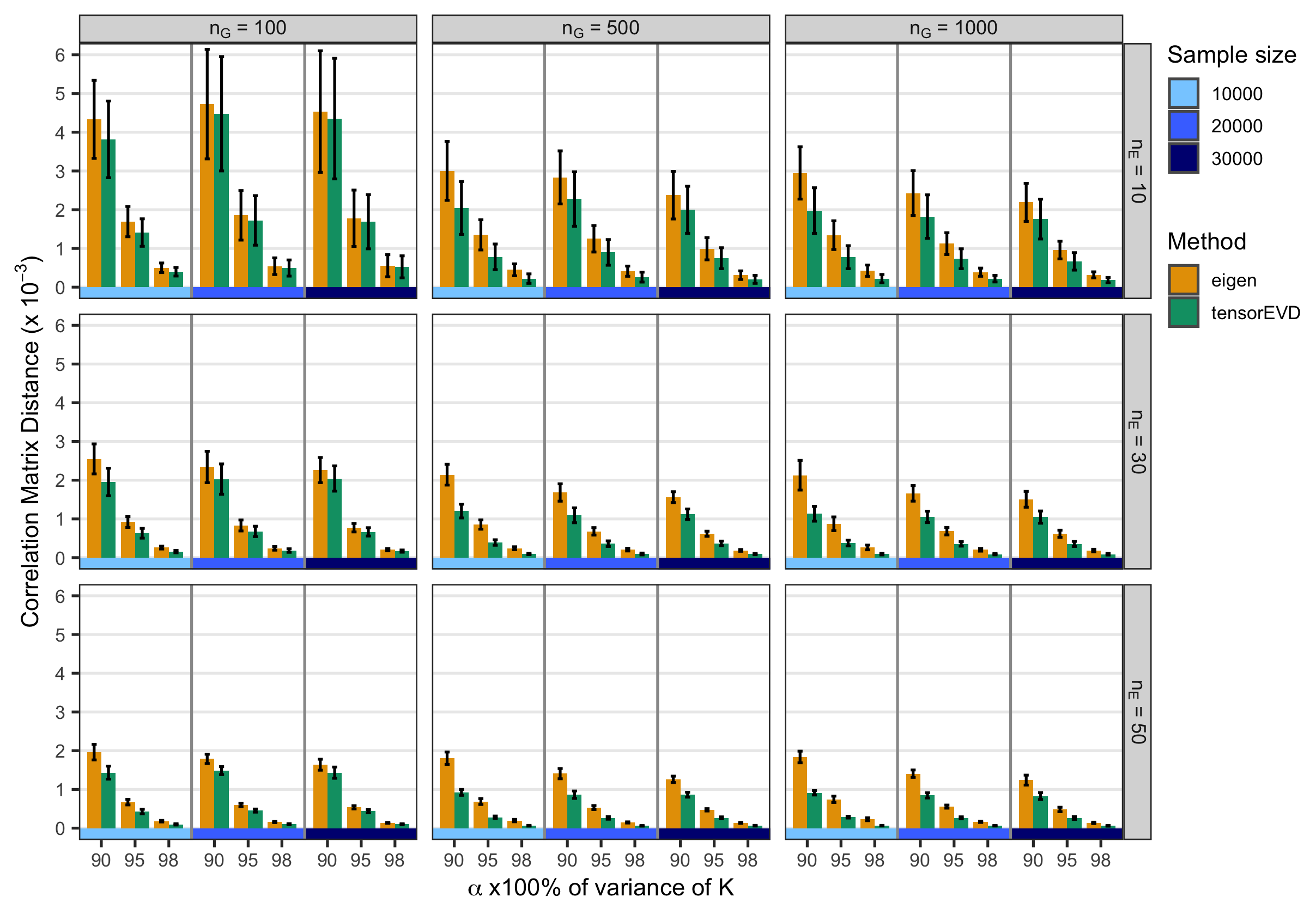


**Supplementary Figure 5.** Correlation Matrix Distance (average $\pm$ SD across 20 replicates) between the Hadamard matrix $\mathbf{K}$ and the approximation (${\hat{\mathbf{K}}}_{\alpha}$) provided by the *eigen* and *tensorEVD* procedures, by sample size ($n=10,000, 20,000,$ and $30,000$) and proportion $\alpha$ of variance of $\mathbf{K}$ explained ($\alpha=0.90, 0.95,$ and $0.98$). Each panel represents a combination of number of hybrids ($n_{G}$) and number of environments ($n_{E}$).


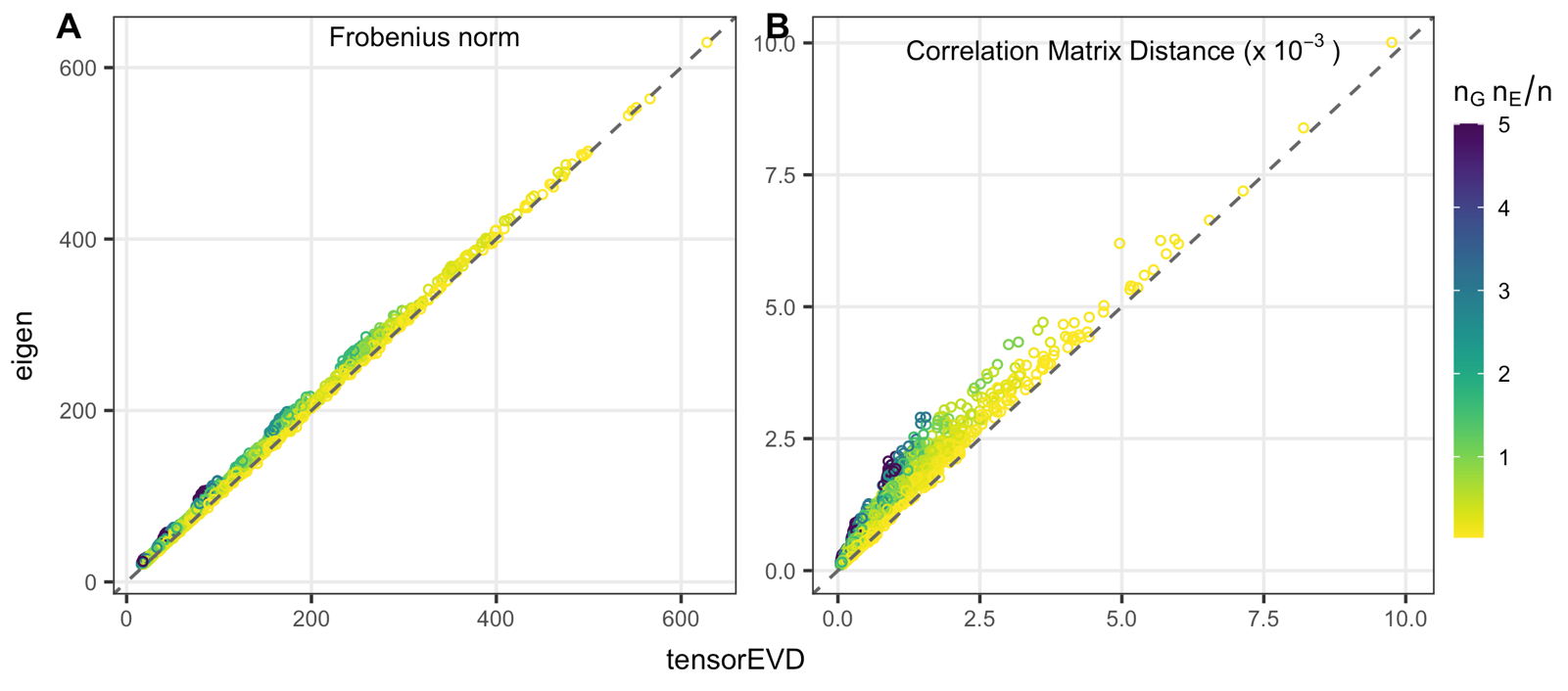


**Supplementary Figure 6.** (**A**) Frobenius norm and (**B**) Correlation Matrix Distance ($\times{10}^{-3}$) between the Hadamard matrix $\mathbf{K}$ and the approximation (${\hat{\mathbf{K}}}_{\alpha}$) provided by the *tensorEVD* (x-axis) and *eigen* (y-axis) procedures. Each point represents a combination of number of hybrids ($n_{G}$), number of environments ($n_{E}$), sample size ($n$), proportion $\alpha$ of variance of $\mathbf{K}$ explained ($\alpha$), and replicate ($1,\ldots,20$). Points are colored according to the ratio between the product of number of hybrids with number of environments, and sample size, $n_{G}n_{E}/n$.


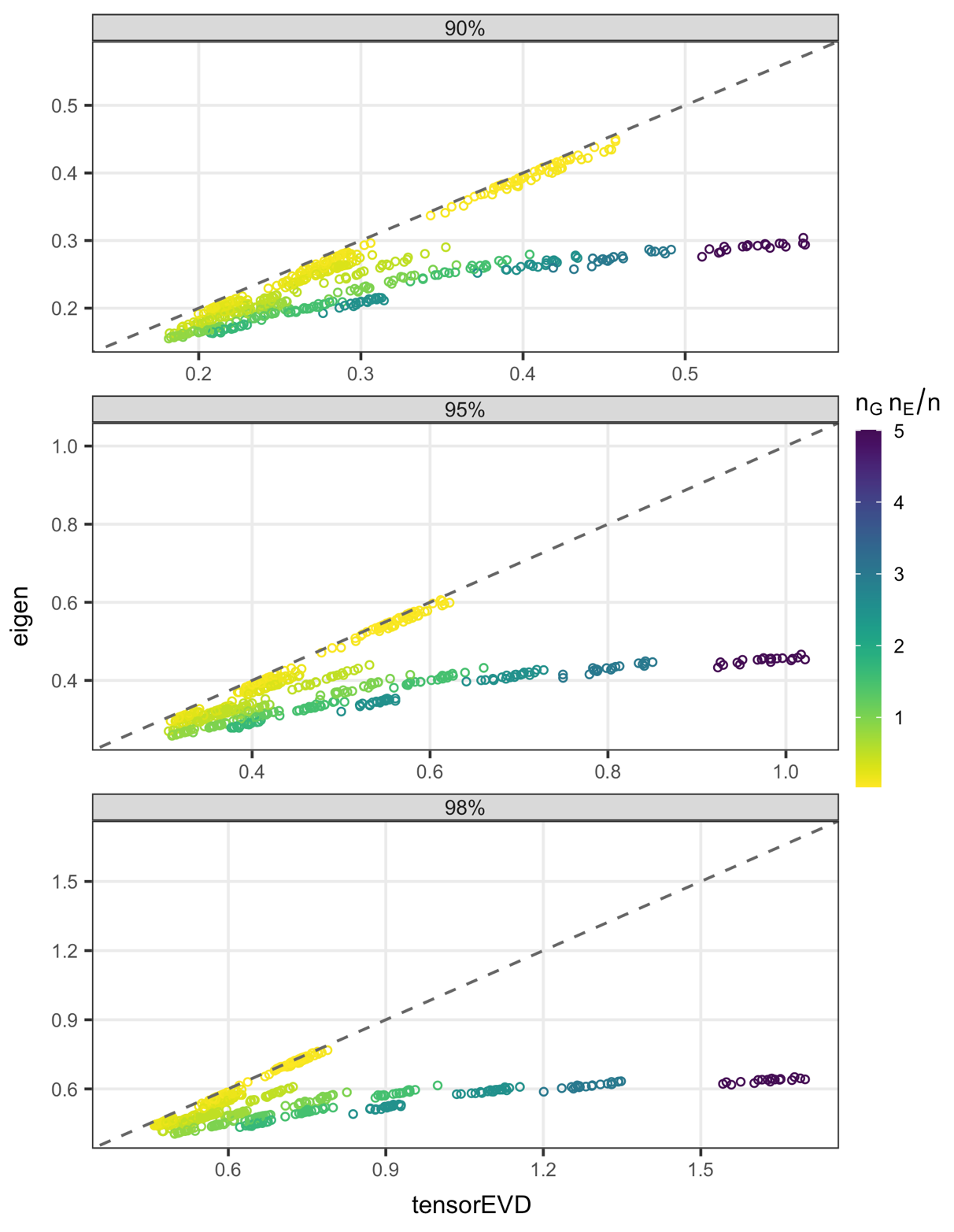


**Supplementary Figure 7.** Number of eigenvectors produced by the *tensorEVD* (x-axis) and *eigen* (y-axis) methods, relative to the rank of matrix **K**. Each point represents a combination of number of hybrids ($n_{G}$), number of environments ($n_{E}$), sample size ($n$), and replicate ($1,\ldots,20$). Points are colored according to the ratio between the product of number of hybrids with number of environments, and sample size, $n_{G}n_{E}/n$. Each panel represents an $\alpha$-value (i.e., proportion of variance of **K** explained, $\alpha=0.90, 0.95,$ and $0.98$).


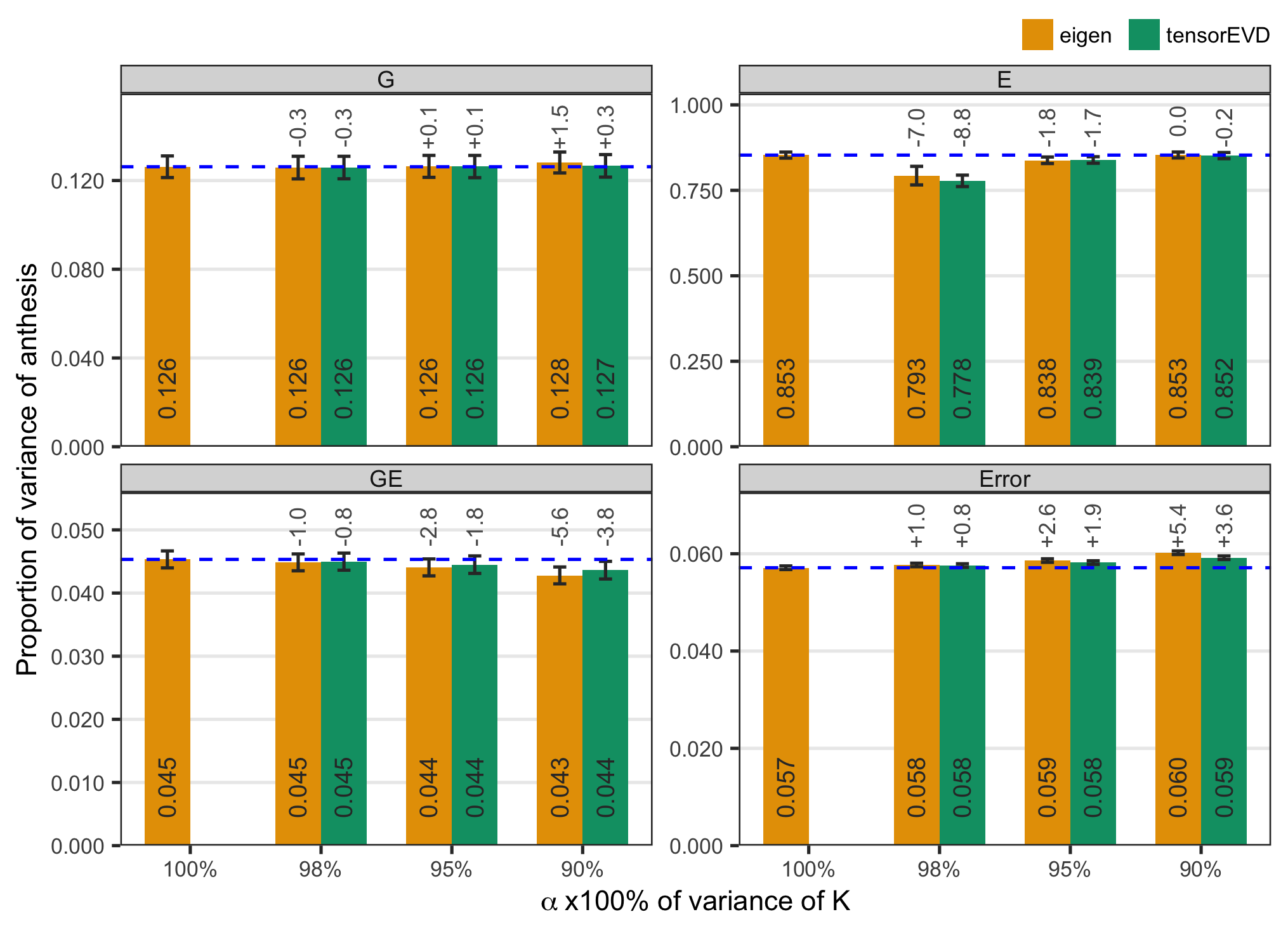


**Supplementary Figure 8.** Proportion of the phenotypic variance (average $\pm$ SD across 5 replicates) of anthesis explained by each model term (G, E, GE, Error) in Equation (3) in the manuscript. The EVD of the Hadamard matrix **K** (covariance matrix of term GE) was performed using eigen and tensorEVD methods for different $\alpha$-values ($\alpha=1.00, 0.98, 0.95,$ and $0.90$). Numbers on the top represent the percentage of change (%) relative to the variance explained by each term in the model that uses full information in **K** (i.e., $\alpha=1.00$) obtained with the eigen method (horizontal dotted line).


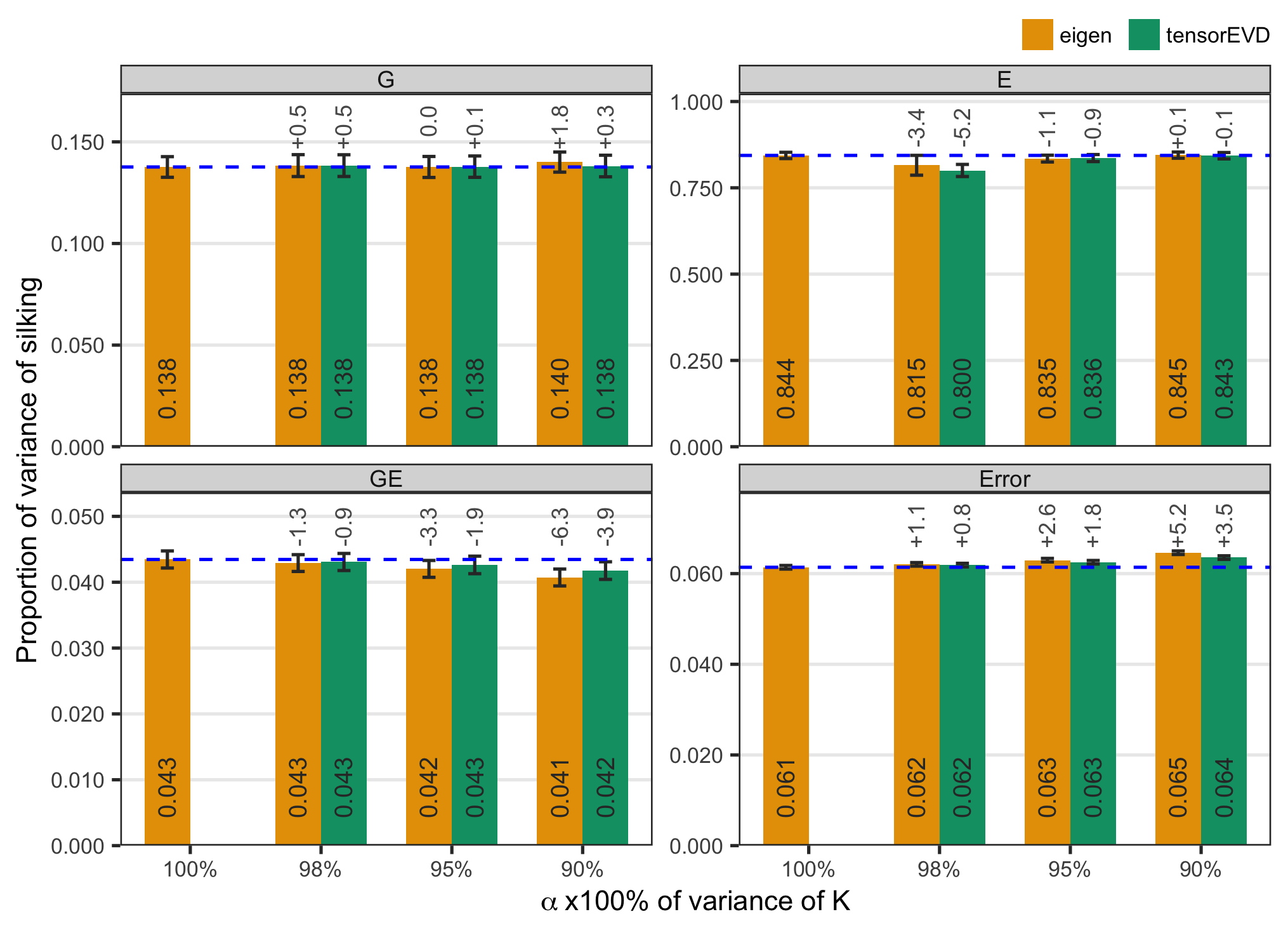


**Supplementary Figure 9.** Proportion of the phenotypic variance (average $\pm$ SD across 5 replicates) of silking explained by each model term (G, E, GE, Error) in Equation (3) in the manuscript. The EVD of the Hadamard matrix **K** (covariance matrix of term GE) was performed using eigen and tensorEVD methods for different $\alpha$-values ($\alpha=1.00, 0.98, 0.95,$ and $0.90$). Numbers on the top represent the percentage of change (%) relative to the variance explained by each term in the model that uses full information in **K** (i.e., $\alpha=1.00$) obtained with the eigen method (horizontal dotted line).


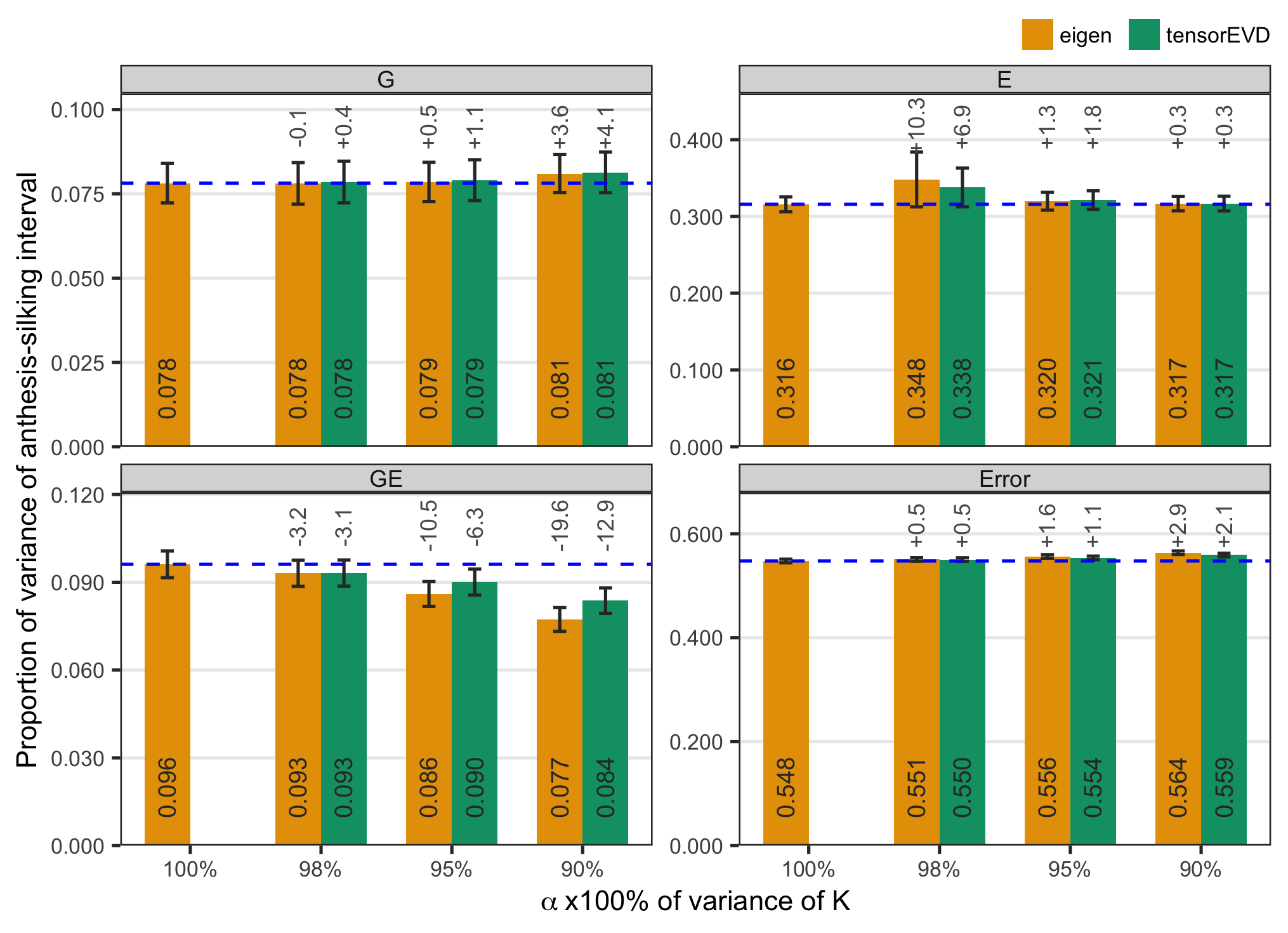


**Supplementary Figure 10.** Proportion of the phenotypic variance (average $\pm$ SD across 5 replicates) of anthesis-silking interval explained by each model term (G, E, GE, Error) in Equation (3) in the manuscript. The EVD of the Hadamard matrix **K** (covariance matrix of term GE) was performed using eigen and tensorEVD methods for different $\alpha$-values ($\alpha=1.00, 0.98, 0.95,$ and $0.90$). Numbers on the top represent the percentage of change (%) relative to the variance explained by each term in the model that uses full information in **K** (i.e., $\alpha=1.00$) obtained with the eigen method (horizontal dotted line).


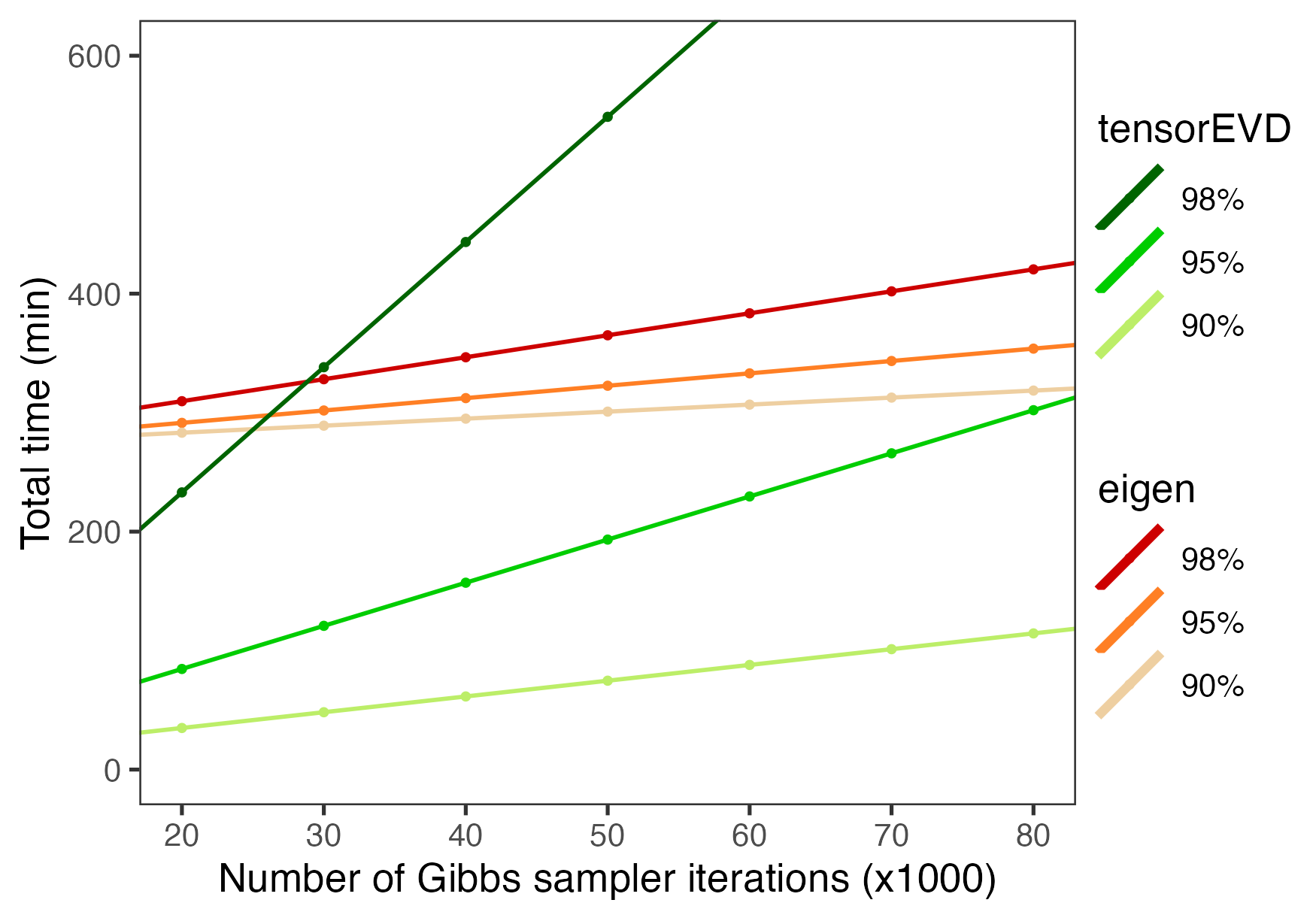


**Supplementary Figure 11.** Total computation time (eigenvalue decomposition + model fitting) versus number of iterations of the Gibbs sampler, for eigen and tensorEVD methods by $\alpha$-values ($\alpha=0.98, 0.95,$ and $0.90$). Each line (representing a combination of a method and $\alpha$-value) was obtained using a regression with slope equal to the average time per iteration (column 5) and intercept equal to the computation time of the EVD (column 4) plus an over-heading time (matrices preparation and hyperparameters setting), taken from Table 1. The over-heading time was calculated as the difference between the total time of the Gibbs sampling (column 6) and the time of implementing the iterations, calculated as $time\_per\_iter\times50,000/60$.

**References**

Golub G. H., and C. F. Van Loan. 1996. *Matrix computatitions*. Johns Hopkings University, Baltimore, MD.

Herdin M., N. Czink, H. Özcelik, and E. Bonek. 2005. Correlation Matrix Distance, a Meaningful Measure for Evaluation of Non-Stationary MIMO Channels. In: Guarente L, Hyslop L, editors. *IEEE 61st Vehicular Technology Conference*. New York: IEEE. p. 136–140

Lima D. C., J. D. Washburn, J. I. Varela, Q. Chen, J. L. Gage, *et al.* 2023. Genomes to Fields 2022 Maize genotype by Environment Prediction Competition. BMC Res. Notes 16: 148.

Lopez-Cruz M., F. Aguate, J. Washburn, S. K. Dayane, C. Lima, *et al.* 2023. Leveraging Data from the Genomes to Fields Initiative to Investigate genotype-by-environment interactions in Maize in North America. Nat. Commun. 14: 6904.

Searle S. R. 1982. *Matrix Algebra Useful for Statistics*. New Jersey: John Wiley & Sons, Inc

VanRaden P. M. 2008. Efficient methods to compute genomic predictions. J. Dairy Sci. 91: 4414–4423.
